# Bone Marrow-Targeted Liposomes Loaded with Bortezomib Overcome Multiple Myeloma Resistance

**DOI:** 10.1021/acsnano.4c10597

**Published:** 2025-03-21

**Authors:** Rotem Menachem, Igor Nudelman, Avital Vorontsova, Ido Livneh, Mor Sela, Madeleine Benguigui, Bar Manobla, Yael Shammai, Abhilash Deo, Chen Buxbaum, Ron Bessler, Ziv Raviv, Jeny Shklover, Josué Sznitman, Aaron Ciechanover, Avi Schroeder, Yuval Shaked

**Affiliations:** † Department of Cell Biology and Cancer Science, Rappaport Faculty of Medicine, Technion−Israel Institute of Technology, Haifa 3525422, Israel; ‡ Rappaport Technion Integrated Cancer Center, Technion − Israel Institute of Technology, Haifa 3525422, Israel; § Faculty of Chemical Engineering, Technion − Israel Institute of Technology, Haifa 3200003, Israel; ∥ Faculty of Biomedical Engineering, Technion−Israel Institute of Technology, Haifa 3200001, Israel

**Keywords:** liposomes, multiple myeloma, resistance, bortezomib, SDF-1-CXCR4 axis

## Abstract

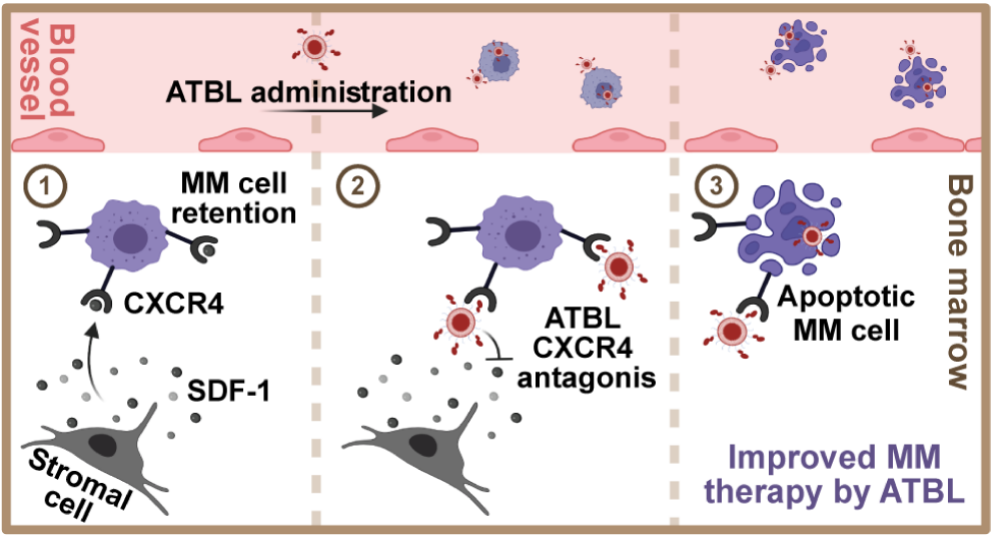

Multiple myeloma (MM) poses a significant therapeutic challenge due to its persistent progression and low survival rate. Although the proteasome inhibitor bortezomib has revolutionized MM treatment, MM aggressiveness and drug resistance remain critical concerns. To tackle this problem, we developed AMD3100-targeted Bortezomib Liposomes (ATBL) designed for the targeted delivery of bortezomib to MM cells. Uptake of ATBL into MM cells was dependent on CXCR4 and was enhanced compared to nontargeted liposomes, both *in vitro* and *in vivo*. Treating MM-bearing mice with ATBL achieved superior therapeutic efficacy compared to treatment with free bortezomib or nontargeted bortezomib-loaded liposomes. Notably, the therapeutic activity of ATBL was limited in mice inoculated with CXCR4-knockdown MM cells, highlighting CXCR4 as a potential biomarker for ATBL response. Importantly, ATBL was effective against an aggressive and bortezomib-resistant MM clone both *in vitro* and *in vivo*. Toxicity and biodistribution profiles demonstrated the safety and bone marrow-targeting ability of ATBL. Collectively, this study highlights ATBL as a promising next-generation proteasome inhibitor-based therapy that incorporates bone marrow-targeting ability and sensitizing elements to overcome drug resistance in MM.

## Introduction

Multiple myeloma (MM) constitutes approximately 2% of cancer-related mortalities and represents the third most prevalent hematologic malignancy globally.^1^ This disease is characterized by the clonal expansion of plasma cells primarily within the bone marrow (BM) microenvironment.^2^ Despite advancements in therapeutic modalities, MM remains an incurable disease.^1^ The proteasome inhibitor, bortezomib (BTZ), is a cornerstone in MM management. It is often utilized alongside the immunomodulatory agent, thalidomide, demonstrating significant efficacy.^3^ The integration of BTZ into treatment regimens has augmented patient survival rates, currently showing a median overall survival of 5 years.^4^ Nevertheless, a subset of MM patients exhibits resistance to initial therapeutic interventions.^5^ Even with the development of second-generation BTZ derivatives or alternative combinatorial therapies encompassing chemotherapy and targeted agents in subsequent lines of therapy, acquired resistance often develops, resulting in clinical deterioration and increased mortality rates.^1,5^ Similar to other anticancer drugs, multidrug resistance (MDR) mechanisms are the main causes of decreased BTZ therapeutic activity.^6^ These include decreased drug uptake, changes in cell metabolic signature, inhibition of MM apoptosis, cell cycle dysfunction, and increased drug excretion.^7^ In addition to these tumor-intrinsic resistance mechanisms, the host response to BTZ therapy induces pro-tumorigenic effects that support MM aggressiveness and disease expansion, as demonstrated by our previous studies.^8,9^ Collectively, these findings highlight the multifaceted nature of MM aggressiveness and resistance following therapy, and the need for novel drugs to overcome these obstacles.

Drug resistance may also arise from pharmacological constraints such as inadequate targeting ability, limited solubility, poor bioavailability, and systemic toxicity.^10^ These constraints may be mitigated by nanoparticle-based therapies. For example, drug-loaded liposomes offer numerous advantages such as improved drug stability, controlled release of drugs, increased systemic circulation times, and reduced off-target toxicities.^11^ The passive accumulation of drug-loaded nanoparticles is facilitated by the enhanced permeability and retention (EPR) effect in tumors, although its effectiveness is somewhat restricted in certain tissues, such as the bone marrow, due to potential physical barriers.^12,13^ Relying solely on passive targeting strategies may not fully exploit the efficacy potential of liposome-based drug delivery systems to the bone. However, active targeting strategies involving the conjugation of targeting molecules onto nanocarrier surfaces, can potentially enhance their affinity to tumor tissues, thereby facilitating more precise uptake by targeted cells. This targeted drug delivery approach has shown significant potential in enhancing the effectiveness of cancer therapies, potentially overcoming drug resistance mechanisms observed in various malignancies.^14−16^

CXCR4, the chemokine receptor for SDF-1 (CXCL12), has a crucial role in maintaining hematopoietic stem cells (HSCs) within the bone-marrow microenvironment.^17^ As such, the FDA-approved CXCR4 antagonist, AMD3100 (AMD) (Mozobil), commonly utilized for HSC mobilization prior to bone-marrow transplantation, has a significant impact on bone-marrow function.^18^ Notably, CXCR4 is selectively overexpressed on some MM cells, with 60% of patients displaying high levels of CXCR4 on MM cells.^19,20^ While previous studies have explored the synergistic effects of AMD and BTZ in MM therapy,^21,22^ and the potential of CXCR4 antagonist liposomal drug delivery systems,^23^ here we present an approach integrating these different aspects. As opposed to previous studies demonstrating electrostatic absorbance of AMD to liposome surface,^24^ we designed a liposome with covalently conjugated AMD directly to its surface, while simultaneously encapsulating BTZ within it. These liposomes, termed AMD-Targeted Bortezomib Liposomes (ATBL), were constructed using a well-established, FDA-approved lipid composition and employed a straightforward synthesis method to attach the targeting molecule. This approach provides a significant clinical advantage that integrates innovation with regulatory compliance and ease of manufacturing. We first characterized ATBL affinity and activity *in vitro* by evaluating its uptake by MM cells expressing various levels of CXCR4. Subsequently, we validated ATBL therapeutic activity in mouse models of MM, and studied its biodistribution and toxicity profile. Importantly, our study demonstrates the effectiveness of ATBL against BTZ-resistant and aggressive MM tumors. This preclinical study serves as a proof-of-concept for the prospective development of ATBL as a therapeutic intervention for aggressive MM.

## Results

### The Generation and Chemical Characterization of ATBL

**Figure 1:**
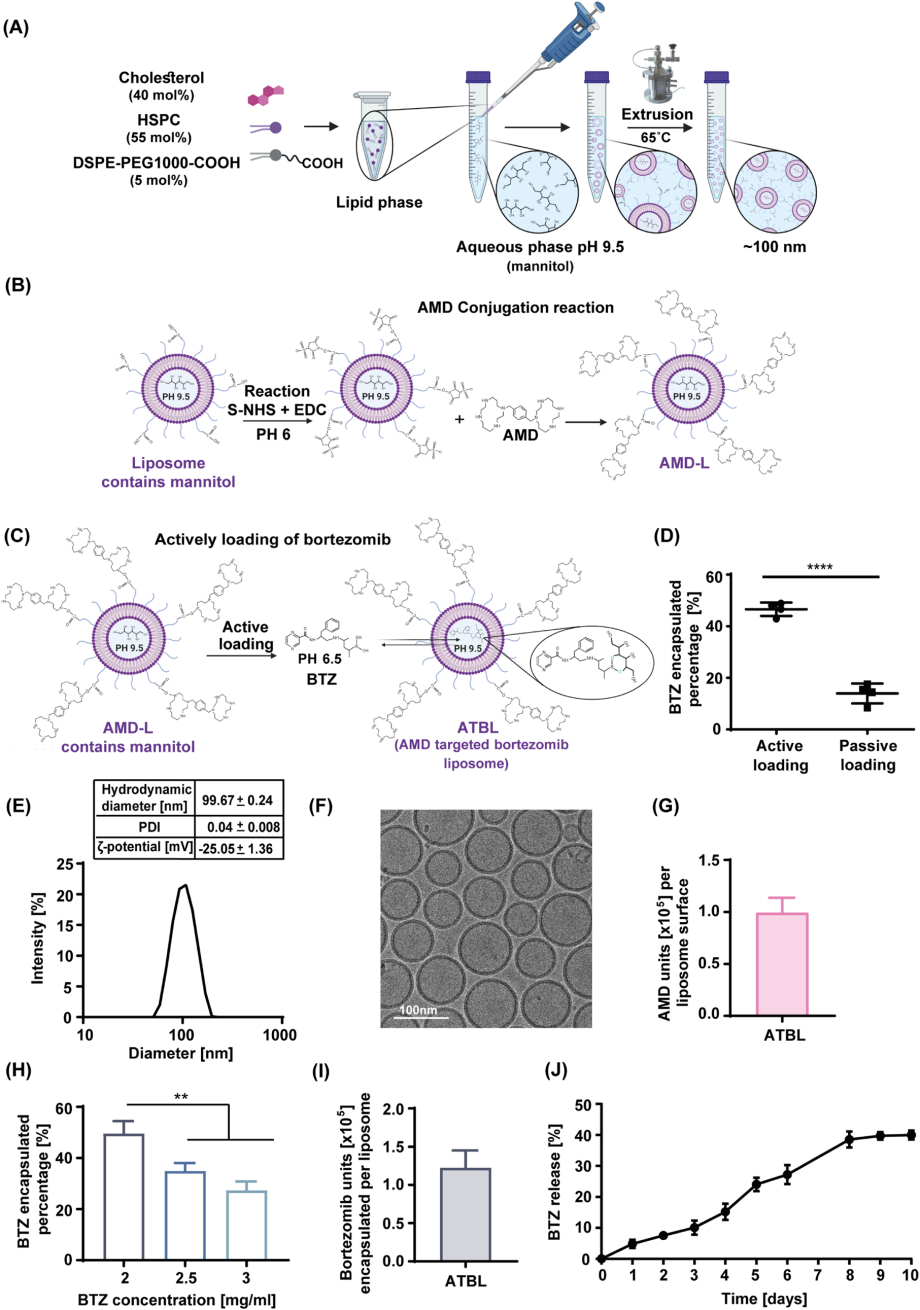
Synthesis of AMD-conjugated, BTZ-loaded liposomes designed for targeting multiple myeloma cells in the bone marrow. (A) A schematic illustration of liposome generation via the ethanol injection method into aquatic phase of acetic acid and mannitol at pH = 9.5 followed by extrusion to ensure a size of ∼ 100 nm. (B) A schematic illustration showing AMD conjugation to the liposomes using EDC and S-NHS reaction. (C) A schematic illustration showing active loading of BTZ into liposomes using pH and concentration gradients. (D) A comparison of BTZ encapsulation into liposomes via passive versus active loading (*n* = 4). (E) ATBL hydrodynamic diameter, PDI and surface electrostatic ζ-potential were characterized by dynamic light scattering using Zetasizer. (F) ATBL imaging for size and shape using cryogenic transmission electron microscopy (cryo-TEM). (G) Quantification of AMD molecules conjugated to the liposome surface (*n* = 3), as described in Materials and Methods. (H) Encapsulation efficiency (%EE) of BTZ was tested at various BTZ concentrations as indicated in the figure (*n* = 3/group). (I) Quantification of BTZ molecules encapsulated per liposome (*n* = 4), as described in Materials and Methods. (J) A profile of BTZ release was conducted at 37 °C and in 50% serum (*n* = 3). Results are presented as mean ± SD. Two-tailed unpaired Student’s *t* test was used for the statistical analysis in C, and One-way ANOVA was used for the statistical analysis in H, with multiple comparisons test, adjusted p-value; **p < 0.01, ****p < 0.0001. BTZ, bortezomib; AMD, AMD3100; AMD-L, AMD liposomes; S-NHS, *N*-hydroxysulfosuccinimide sodium salt; EDC, *N*-(3- dimethylaminopropyl)-N’-ethyl carbodiimide hydrochloride; PDI, particle size distribution.

To enable efficient and targeted delivery of therapeutic BTZ-loaded liposomes to MM cells within the bone-marrow, we developed AMD-targeted-BTZ-Liposomes (ATBL) in which AMD was conjugated to the outer surface of the liposomes. To ensure ATBL stability and clinical compatibility, the liposomes contained HSPC, cholesterol and DSPE-PEG1000-COOH (Figure S1A), adhering to the composition of liposomes currently in clinical use for treating cancer.^25^ Following liposome preparation via the ethanol injection method,^26^ AMD was conjugated to carboxylic functionalized PEG extending from the outer surface of the liposomes using EDC carbodiimide cross-linking reaction with S-NHS^27^ (Figure 1A,B). Subsequently, BTZ was actively loaded into the liposomes, using a concentration gradient of mannitol and acetic acid^28,29^ and a pH gradient to ensure the reaction occurred solely inside the liposomes^30^ (Figure 1C). Of note, BTZ active loading was more efficient than passive loading^31^ (Figure 1D). The resulting ATBL exhibited an average hydrodynamic diameter of 99.67 ± 0.24 nm, and particle size distribution (PDI) of 0.04 ± 0.008 with surface electrostatic ζ-potential of −25.05 ± 1.36 mV. The average liposome particle concentration was found to be 1.66 ± 0.37 × 10^13^ particles/ml (Figures 1E and S1B). In addition, ATBL particles were of similar size and shape, as observed by cryo-TEM imaging (Figure 1F).

Conjugation of AMD to the liposome surface was characterized using various methods. First, thin-layer chromatography (TLC) confirmed AMD conjugation (Figure S1C). Second, covalent binding of AMD to the liposomes was estimated by measuring the liposome surface charge. Increasing concentrations of AMD were added to empty nontargeted liposome (EMPTY-L) (zeta potential of −46.24 mV) and the decrease in zeta potential was measured using Zetasizer. The zeta potential of ATBL after dialysis (−25.02 mV) indicates successful conjugation of AMD, which was in the range of 0.1–0.625 mg/mL (Figure S1D). Third, the quantity of AMD molecules conjugated to the liposome surface was found to be 9.92 ± 1.46 × 10^4^/liposome, as measured using the ninhydrin reaction (Figure 1G).

BTZ encapsulation was quantified by optical density. For this, absorbance was measured over a range of wavelengths (230–1000 nm) for serially diluted BTZ-loaded liposomes to identify the optimal detection wavelength of BTZ-loaded liposomes. The maximal absorbance was found to be 325 nm, further validated by a calibration curve of known BTZ concentrations (Figure S1E,F). Subsequently, to investigate the effect of BTZ concentration on encapsulation efficiency (%EE), increasing concentrations of BTZ (2, 2.5, and 3 mg/mL) were added to the AMD-liposomes (AMD-L) by active loading. A significantly higher %EE was observed when liposomes were incubated with 2 mg/mL (49.5%) compared to 2.5 and 3 mg/mL (43.6% and 27.3%, respectively) of BTZ. This suggests that despite its lower concentration, 2 mg/mL of BTZ achieves superior %EE compared to the other concentrations tested (Figure 1H). Therefore, the maximum number of encapsulated BTZ molecules measured in ATBL was 1.22 ± 0.23 x10^5^ /liposome (Figure 1I). We next sought to determine the drug release profile of ATBL. We found a 40% release rate of BTZ from liposomes at 37 °C in 50% serum over a 10-day period (Figure 1J). Furthermore, ATBL showed no significant change in particle size (99.67 nm ± 0.29) and PDI (0.042 ± 0.009) over 12 weeks (Figure S1G,H).

### ATBL Uptake by MM Cells Is Dependent on CXCR4

**Figure 2:**
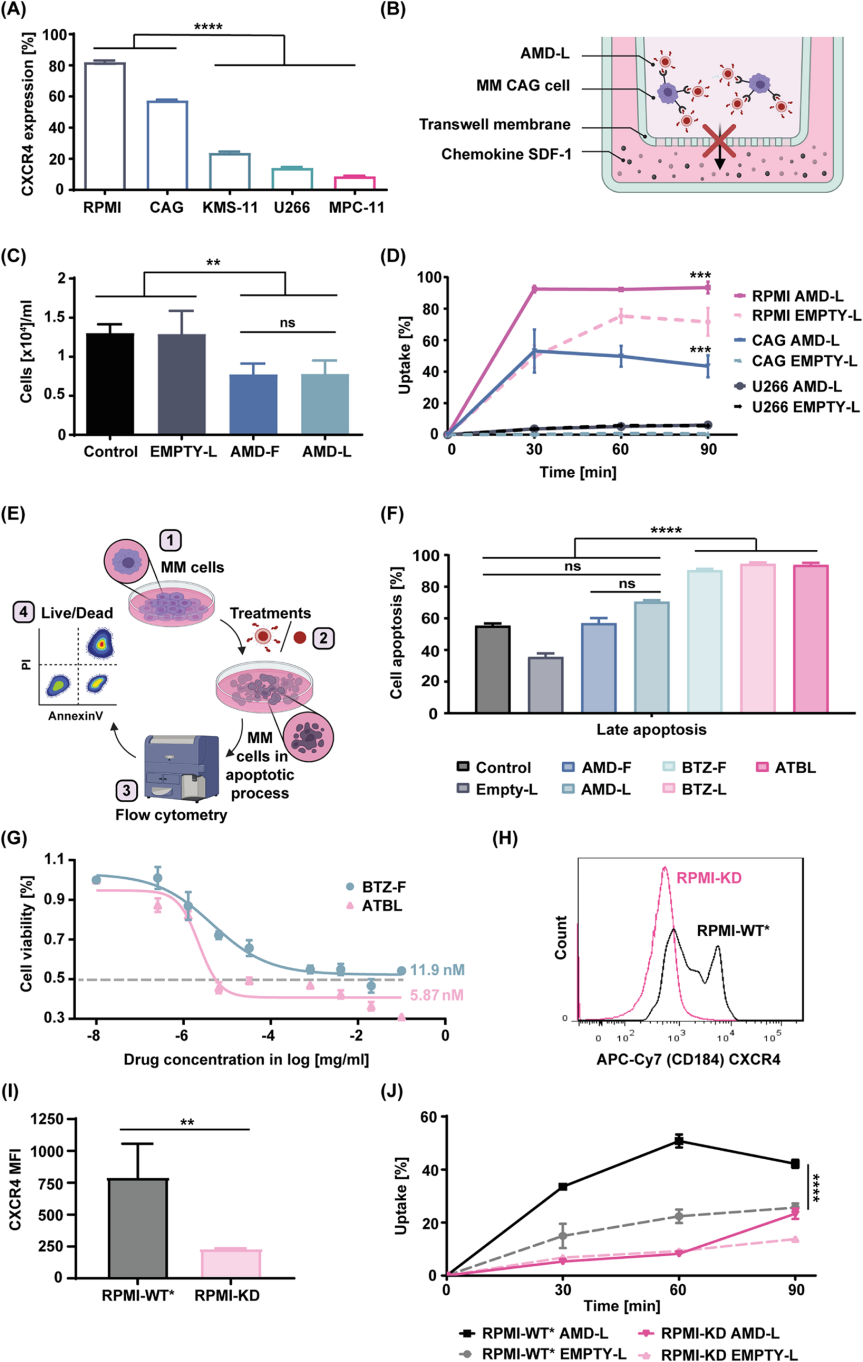
ATBL uptake is CXCR4 dependent. (A) CXCR4 expression in the indicated MM cell lines was evaluated using flow cytometry (*n* = 3/cell line). (B) A schematic illustration of the transwell migration assay in which MM cell migration in response to SDF-1 chemokine is assessed in the presence of AMD-L. (C) CAG cells were treated with AMD-L (1.62 ± 0.35 × 10^12^ liposomes/ml), AMD-F (25 μM), EMPTY-L (1.52 ± 0.96 × 10^12^ liposomes/ml) or control for 90 min. Migration of the treated cells was assessed by the transwell assay at the 24 h time point (*n* = 5/group). (D) *In vitro* cellular uptake of Cy5 labeled AMD-L (4.86 ± 1.04 × 10^11^ liposomes/ml) versus EMPTY-L (4.56 ± 1.19 × 10^11^ liposomes/ml) was assessed in RPMI, CAG and U266 MM cell lines by flow cytometry (*n* = 3/group). (E) A schematic illustration of the 4-step procedure to evaluate MM cell apoptosis in the presence of different treatments. (F) The late apoptosis profile of RPMI cells treated for 48 h with the indicated types of liposomes was assessed by flow cytometry (*n* = 3/group). (G) Viability of RPMI MM cells was assessed in the presence of increasing concentrations of BTZ-F and ATBL. IC50 measurements per treatment are indicated (*n* = 3/group). (H–I) CXCR4 expression on RPMI cells knocked down for CXCR4 (RPMI-KD) and their wild-type counterpart (RPMI-WT*) was assessed by flow cytometry. A representative histogram is shown in H, and mean fluorescence intensity (MFI) is shown in I (*n* = 3–4/group). (J) *In vitro* cellular uptake of rhodamine labeled AMD-L (4.86 ± 1.04 × 10^11^ liposomes/ml) compared to EMPTY-L (4.56 ± 1.19 × 10^11^ liposomes/ml) was assessed in MM RPMI-KD and RPMI-WT* cells by flow cytometry (*n* = 3/group). Results are presented as mean ± SD. One-way ANOVA was used for the statistical analysis in A, C and F with multiple comparisons test. Two-way ANOVA was used for the statistical analysis in D and J with multiple comparisons test. Two-tailed unpaired Student’s *t* test was used for the statistical analysis in I, adjusted p-value; **p < 0.01, ***p < 0.001, ****p < 0.0001. ns; not significant; MM, multiple myeloma; RPMI, RPMI8226; RPMI-KD, RPMI8226 CXCR4 knockdown cells; RPMI-WT*, RPMI8226 wild type cells that underwent the same genetic manipulation as the RPMI-KD cells, but without the inclusion of the guide RNA; IC50, half maximal inhibitory concentration; ATBL, AMD targeted bortezomib liposomes; BTZ-F, bortezomib free drug; BTZ-L, bortezomib liposomes; AMD-F, AMD3100 free drug; AMD-L, AMD3100 liposomes; EMPTY-L, empty nontargeted liposome; Control, vehicle.

AMD binds exclusively to CXCR4,^32^ a receptor that exhibits heterogeneous expression on MM cells.^19^ Despite this variability, studies have shown that 60% of patients with MM exhibit CXCR4-positive MM cells,^20^ highlighting the potential of ATBL for targeted drug delivery. To determine whether ATBL binds to MM cells and exerts a therapeutic effect *in vitro*, we first analyzed the levels of CXCR4, and its ligand SDF-1, in several MM cell lines. We found that CXCR4 is differentially expressed in various MM cell lines, with the human RPMI and CAG MM cell lines displaying the highest percentage of CXCR4 expression (Figures 2A and S2A). In addition, SDF-1 was shown to be secreted by RPMI cells but not by any of the other cell lines tested, in the presence and absence of AMD (Figure S2B).

To test whether AMD-conjugated liposomes bind to CXCR4 on MM cells, we used the migration assay to test chemotaxis. As over 50% of CAG cells express CXCR4, but not the ligand SDF-1, we used this cell line for the assay. To this end, CAG cells were treated with AMD-L or EMPTY-L for 90 min. AMD in its free form (AMD-F) was used as a positive control. Following treatment, the cells were placed in the upper chamber of a transwell plate and migration into the SDF-1-containing lower chamber was quantified (Figure 2B, for illustration). Both AMD-F and AMD-L decreased the number of migrating cells by more than 50% in comparison to EMPTY-L, indicating that AMD-conjugated liposomes bind to CXCR4 (Figure 2C). Next, we assessed the uptake of AMD-L by MM cells displaying varying degrees of CXCR4 expression, in order to determine whether the expression of CXCR4 affects AMD-L uptake. Strikingly, AMD-L uptake correlated with CXCR4 expression in the different MM cell lines, with RPMI cells displaying the highest uptake, CAG cells displaying an intermediate level of uptake, and U266 cells displaying minimal uptake (Figures 2D and S2C). Of note, in RPMI and CAG cells, uptake of EMPTY-L was significantly reduced in comparison to AMD-L, further indicating that uptake is CXCR4-dependent.

To analyze the therapeutic activity of ATBL *in vitro*, we assessed cell apoptosis in RPMI and CAG cells cultured with ATBL, free BTZ (BTZ-F), BTZ-loaded liposomes (BTZ-L), AMD-F, AMD-L and additional controls (Figure 2E, for illustration). Treatment with AMD-F or AMD-L did not increase cell apoptosis relative to untreated or EMPTY-L controls in both RPMI and CAG cell lines. However, the presence of BTZ in any of the three forms significantly induced cell apoptosis to almost 100% (Figures 2F and S2D). Of note, the concentration of AMD in our experimental system was 25 μM, which is lower than the therapeutic dose described in some preclinical studies.^33,34^ The consistent therapeutic effect observed across these treatments suggests that BTZ is an effective treatment *in vitro*, and that liposomes, whether conjugated or not, do not interfere with its apoptotic potential. Importantly, evaluating the half maximal inhibitory concentration (IC50) of ATBL versus BTZ-F in RPMI cells indicated that ATBL is 2-fold more potent than BTZ-F in killing MM cells (Figure 2G).

To directly investigate the requirement of CXCR4 expression for ATBL therapeutic activity, we used CRISPR-Cas9 to downregulate CXCR4 expression in RPMI cells (RPMI-KD). A wildtype counterpart (RPMI-WT*) was generated using the same genetic manipulation but excluding the guided RNA (Figure S3A, for illustration). Characterization of the two cell lines (Figure S3B, for illustration) demonstrated higher proliferation and migration rates in RPMI-KD compared to RPMI-WT* (Figure S3C–F). Downregulation of CXCR4 expression (Figure 2H,I) corresponded with decreased cell migration in response to SDF-1 (Figure S3G,H), and reduced uptake of AMD-L (Figures 2J and S3I). Furthermore, RPMI-KD and RPMI-WT* cells exhibited similar uptake of EMPTY-L (Figures 2J and S3I). Taken together, these findings highlight ATBL as a potent therapeutic agent against MM cells in vitro, an effect that is dependent on CXCR4 expression.

### ATBL Demonstrates Superior Antitumor Activity against MM Cells Expressing CXCR4 Compared to BTZ

**Figure 3:**
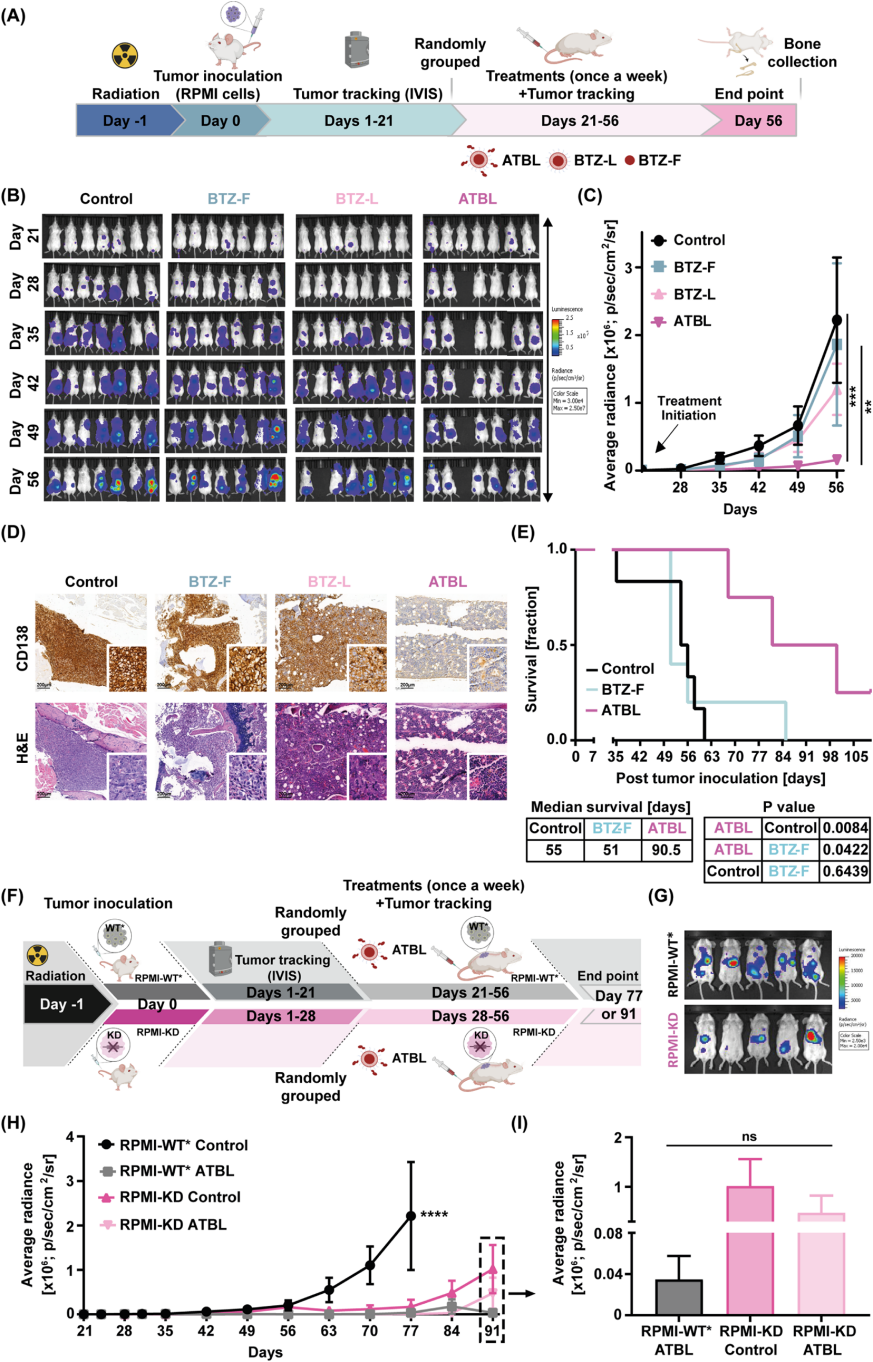
*In vivo* therapeutic effect of ATBL in MM bearing mice. (A) A schematic illustration of the experimental design for evaluating ATBL therapeutic effect *in vivo*: Eight-week-old SCID mice were systemically irradiated (250 rad). After 24 h, MM RPMI cells (5 × 10^6^/mouse) were intravenously injected. On day 21, when sufficient tumor burden was detected by IVIS, mice were intravenously administered with ATBL (1.95 ± 0.43 × 10^13^ liposomes/kg), BTZ-L (1.93 ± 0.43 × 10^13^ liposomes/kg) or control, once a week for a 5-week period. (B–C) Tumor growth and expansion was assessed using the IVIS imaging system on the indicated days (*n* = 5–7 mice/group). (D) At end point, mice were sacrificed, and bone paraffin sections were immunostained with CD138+ (brown) or H&E (Scale bar, 200 μm). (E) In a parallel experiment, SCID mice were treated with ATBL, BTZ-F or control at the same concentrations described in (A) starting on day 28 for 5 weeks. Survival was monitored (*n* = 4–6 mice/group). (F) A schematic illustration of the experimental design: Eight-week-old SCID mice were systemically irradiated (250 rad). After 24 h, RPMI-KD or RPMI-WT* MM cells were intravenously injected (5 × 10^6^/mouse). When sufficient tumor burden was detected by IVIS (day 21 and for RPMI-WT*; day 28 for RPMI-KD), ATBL (1.95 ± 0.43 × 10^13^ liposomes/kg) or control was intravenously administrated once a week for 4 weeks. (G) Representative IVIS images of RPMI-KD and RPMI-WT* tumor bearing mice at 21 and 14 days, respectively, post MM cell inoculation. (H) Tumor growth in mice described in F was assessed using the IVIS imaging system on the indicated days (*n* = 5–6 mice/group). (I) Bar graph indicating tumor size at end point (day 91). Shown are the Kaplan–Meier survival curve, median survival, and p-values using the Wilcoxon statistical test. Results in C and H are presented as mean ± SE. Two-way ANOVA was used for the statistical analysis in C and H with multiple comparisons test. Results in I are presented as mean ± SD. Two-tailed unpaired Student’s *t* test was used for the statistical analysis in I, adjusted p-value; **p < 0.01, ***p < 0.001, ****p < 0.0001. ns, not significant; MM, multiple myeloma; RPMI, RPMI8226; RPMI-KD, RPMI8226 CXCR4 knockdown cells; RPMI-WT*, RPMI8226 wild type cells that underwent the same genetic manipulation as the RPMI-KD cells, but without the inclusion of the guide RNA; ATBL, AMD targeted bortezomib liposomes; BTZ-F, bortezomib free drug; BTZ-L, bortezomib liposomes; Control, vehicle.

To evaluate the therapeutic activity of ATBL *in vivo*, SCID mice bearing RPMI tumors were treated with BTZ-F, BTZ-L, ATBL or vehicle control once a week for a duration of 35 days at the doses indicated in Table S1. Tumor growth was assessed by IVIS, and histopathology was performed at end point (Figure 3A, for illustration). ATBL treatment significantly suppressed tumor growth, an effect that was maintained for over 56 days. In contrast, only minor inhibition of tumor growth was achieved with BTZ-L or BTZ-F compared to the control (Figure 3B,C). Notably, the lack of BTZ-F therapeutic activity is likely due to the relative resistance of RPMI cells to BTZ as well as the frequency at which the drug was administered, i.e., once a week instead of twice a week usually given in mice and patients. To further assess tumor burden, bones obtained at end point were analyzed for expression of the MM surface marker, CD138. Bones of ATBL-treated mice displayed dramatic reduction in CD138 expression compared to control, BTZ-F and BTZ-L groups, further demonstrating the potent therapeutic effect of ATBL (Figure 3D). In a parallel experiment designed to assess survival, comparing ATBL to the clinically relevant BTZ-F drug, SCID mice injected with RPMI MM cells were treated for a period of 35 days with BTZ-F, ATBL or control. ATBL-treated mice exhibited significantly longer survival compared to BTZ-F-treated or control mice, with a median survival of ∼ 90 days compared to the 51–55 days in BTZ-F and control groups (Figure 3E). These results demonstrate that ATBL is an effective treatment option for MM and displays superior antitumor activity compared to BTZ-F.

To further assess whether the therapeutic activity of ATBL is dependent on CXCR4 expression in mice bearing MM cells, we inoculated mice with RPMI-KD or RPMI-WT* cells (Figure 3F, for illustration). Tumor growth assessment by bioluminescence demonstrated that RPMI-KD cells grew much slower than RPMI-WT* control cells. Interestingly, the cell lines displayed differential tumor distribution and growth patterns. RPMI-KD tumor cells were not restricted to the bone marrow, as shown in the RPMI-WT* group, but were also found systemically and in other organs (Figure 3G). This observation suggests that CXCR4 maintains MM cells in the bone, and that the bone is an important supporting niche for MM growth. For this reason, treatment in the RPMI-KD group was initiated a week after the RPMI-WT* group, in order to maintain a fair comparison of ATBL therapeutic activity between the two groups as much as possible. Importantly, while ATBL completely abolished the growth of RPMI-WT* tumors, it demonstrated less effective therapeutic activity against RPMI-KD cells (Figure 3H,I). Reduced ATBL effectiveness is likely due to reduced drug update as well as the fact that tumors are not located solely in the bone marrow but also in other organs. Overall, these findings demonstrate that ATBL is effective against MM cells when the cells express CXCR4.

### ATBL Is Targeted to the Bone and Exhibits Minimal Toxicity

**Figure 4:**
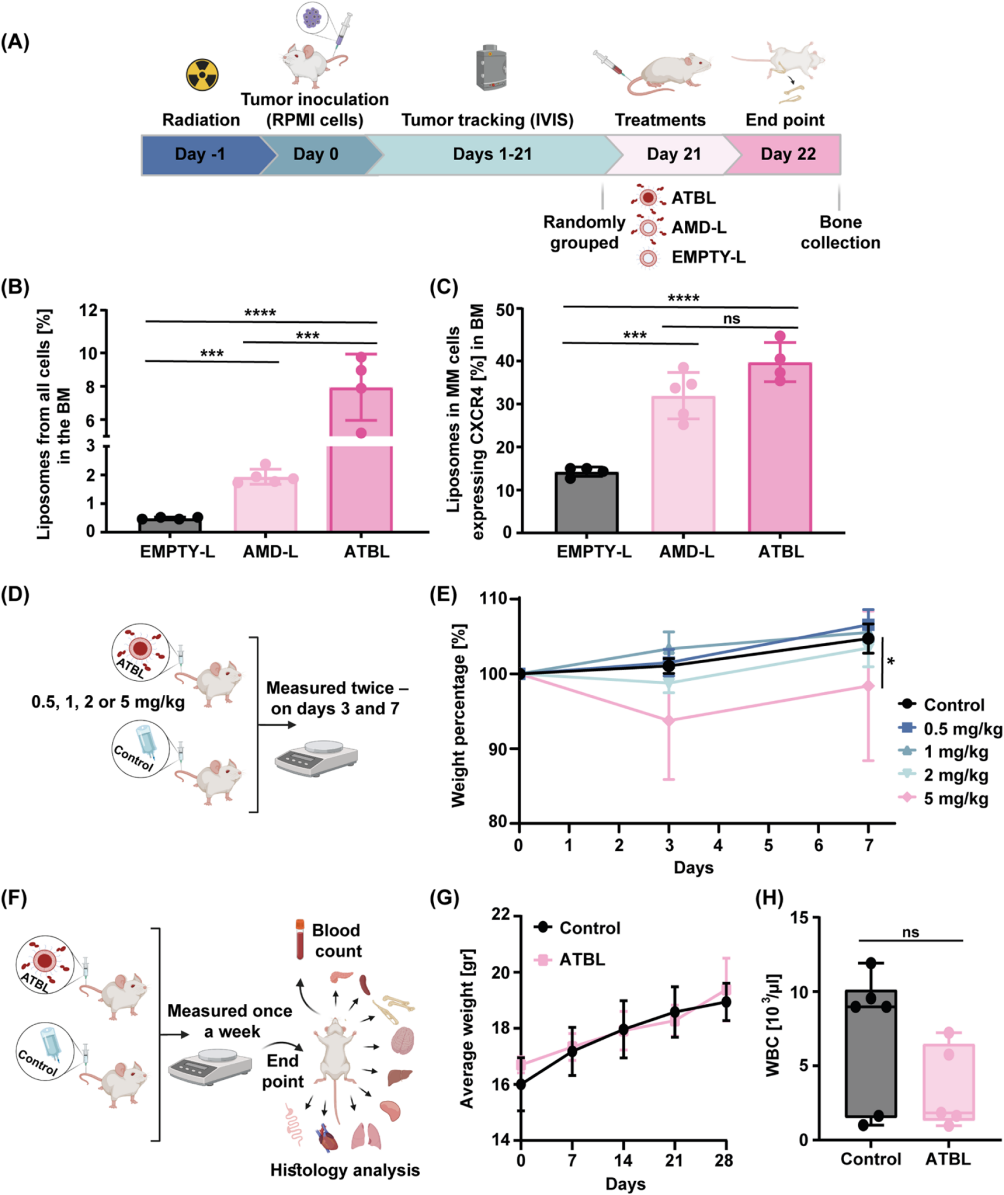
ATBL biodistribution and toxicity. (A) A schematic illustration of the biodistribution experimental design, as detailed in Materials and Methods. Eight-week old SCID mice were systemically irradiated at a dose of 250 rad. After 24 h, MM RPMI cells (5 × 10^6^/mouse) were intravenously injected. On day 21, when sufficient tumor burden was detected by IVIS, rhodamine-labeled ATBL (1.95 ± 0.43 × 10^13^ liposomes/kg), AMD-L (8.10 ± 1.74 × 10^13^ liposomes/kg) or EMPTY-L (7.59 ± 1.98 × 10^13^ liposomes/kg) were intravenously administrated. After 24 h, mice were sacrificed, and bone-marrow (BM) cells were flushed from the bones and analyzed by flow cytometry (*n* = 5 mice/group). (B) The percentage of rhodamine positive cells in the BM is presented. (C) The percentage of rhodamine positive MM cells expressing CXCR4 in the BM is presented. (D) A schematic illustration of dose limiting toxicity experimental design, as detailed in Materials and Methods. (E) The percent in body weight change of mice treated with control or increasing doses of ATBL over a 7-day period. ATBL doses corresponded to equivalent doses of BTZ 0.5, 1, 2, and 5 mg/kg, calculated based on liposome concentrations of 9.77 ± 2.16 × 10^12^, 1.95 ± 0.43 × 10^13^, 3.91 ± 0.86 × 10^13^, 9.77 ± 2.16 × 10^13^ (liposomes/kg), respectively (*n* = 5 mice/group). (F) A schematic illustration of toxicity profiling experimental design, as detailed in Materials and Methods. (G) The average weight of 8 week old BALB/c mice treated with ATBL (1.95 ± 0.43 × 10^13^ liposomes/kg) or control administered once a week for 4-week period (*n* = 5 mice/group) was assessed weekly. (H) The WBC count of mice treated as in G was measured at end point (after 4 weeks of treatment). Results are presented as mean ± SD. Two-tailed unpaired Student’s *t* test was used for the statistical analysis in B and C. Two-way ANOVA was used for the statistical analysis in E and G with multiple comparisons test. One-way ANOVA was used for the statistical analysis in H with multiple comparisons test, adjusted p-value; *p < 0.05, ***p < 0.001, ****p < 0.0001. ns, not significant; BM, bone marrow; MM, multiple myeloma; RPMI, RPMI8226; MTD, maximum tolerated dose; WBC, white blood cell; ATBL, AMD targeted bortezomib liposomes; BTZ-F, bortezomib free drug; AMD-L, AMD3100 liposomes; EMPTY-L, empty nontargeted liposome; Control, vehicle.

CXCR4 is commonly expressed by immune cells and in the bone-marrow compartment, with notably high expression levels observed on MM cells.^35^ To verify that ATBL selectively targets MM cells via interaction with CXCR4, we evaluated its biodistribution in ATBL-treated, MM-bearing mice. To this end, mice were inoculated with RPMI cells and after 21 days were injected with AMD-liposomes encapsulating gadolinium (Gd-AMD-L), prepared 24 h prior to its administration. Organs were collected at various time points postinjection (*i.e*., 4, 12, 24, and 48 h; Figure S4A, for illustration). After the initial drop in gadolinium (Gd) concentration in blood at the 4 h time point, a peak in Gd concentration was observed in liver, lung, and bones at the 24 h time point, indicating liposome accumulation (Figure S4B,C). To further test the distribution of ATBL in the bone, mice were administered with rhodamine-labeled ATBL, AMD-L or EMPTY-L and bones were removed 24 h after liposome administration (Figure 4A, for illustration). Of note, all rhodamine-labeled liposomes were used within 24 h after they were prepared. Both AMD-L and ATBL exhibited significantly increased uptake by bone marrow cells, and specifically RPMI MM cells, compared to EMPTY-L. Notably, ATBL uptake by bone marrow cells was four times higher than that of AMD-L, probably due to rhodamine-positive apoptotic MM cells that underwent phagocytosis by macrophages (Figure 4B,C). These results indicate that AMD indeed functions as a targeting moiety and facilitates liposome accumulation in the bone.

Next, we evaluated the toxicity profile of ATBL based on mouse body weight and white blood cell (WBC) count. We first analyzed the maximum tolerated dose (MTD) of ATBL. To this end, increasing concentrations of ATBL were administered to mice followed by body weight measurements over a week (Figure 4D, for illustration). We found that ATBL is tolerable up to a BTZ equivalent dose of 5 mg/kg, although a marked reduction in body weight was noticeable at this dose (Figure 4E). Of note, the 5 mg/kg dose is 5-fold higher than the therapeutic dose used in the preclinical models in mice.^36^ We then analyzed the toxicity profile of ATBL at a BTZ equivalent dose of 1 mg/kg, given once a week for a 4-week period. This dosage was found to be nontoxic based on body weight measurements and WBC count analyzed at end point (Figure 4F–H). Furthermore, histological analysis of various organs revealed no signs of toxicity during ATBL treatment (Figure S4D). Overall, these results indicate that ATBL is well tolerated.

### ATBL Does Not Induce Host-Mediated Pro-Tumorigenic Activities

**Figure 5:**
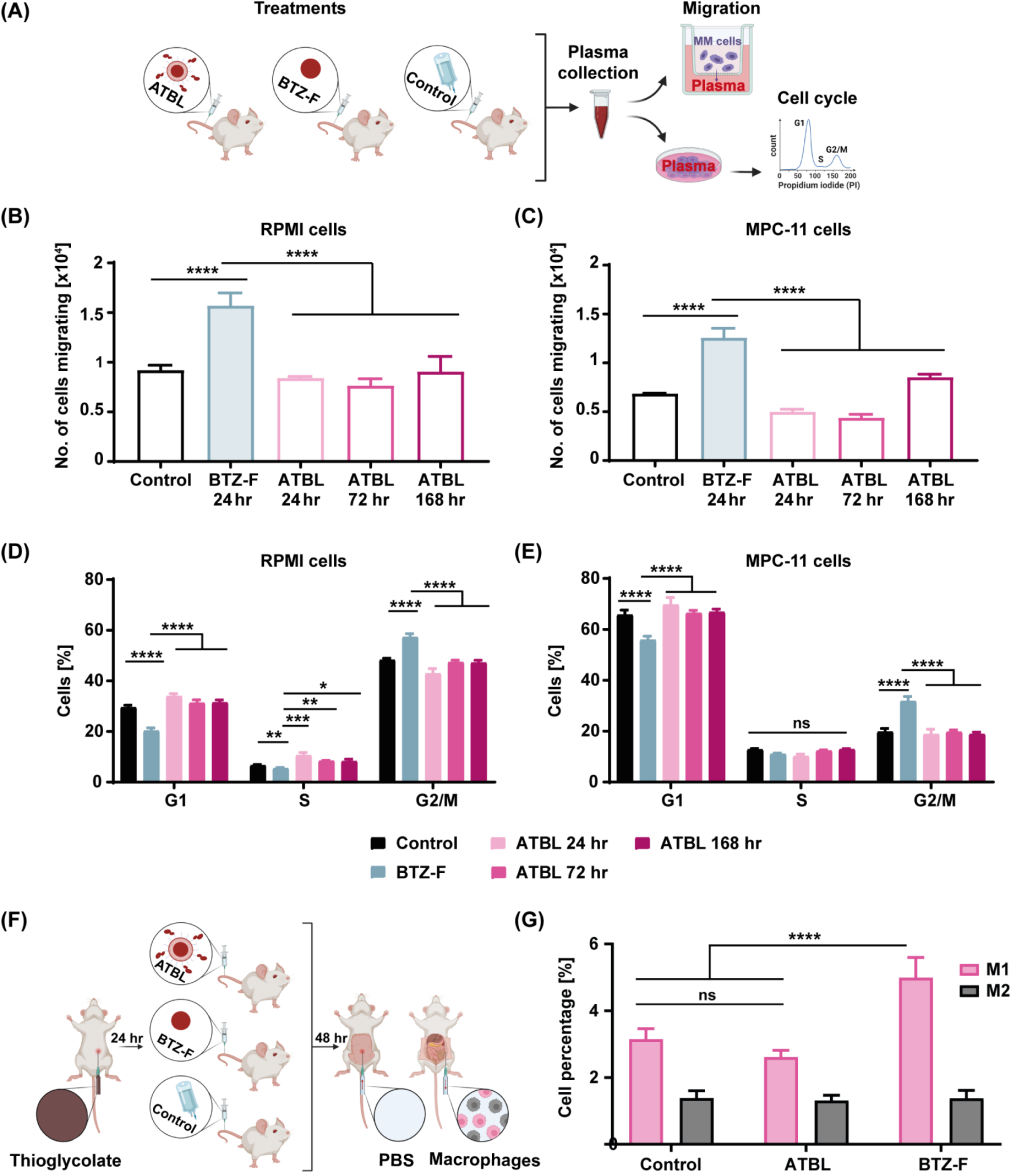
ATBL does not induce host-mediated pro-tumorigenic activities. (A) A schematic illustration of the experimental design for evaluating host-mediated effects *in vitro*: Eight-week-old BALB/c mice were treated with ATBL (1.95 ± 0.43 × 10^13^ liposomes/kg), BTZ-F (1 mg/kg) or control. Plasma was drawn at various time points and used in cell migration and proliferation assays. (B–C) Migration of RPMI and MPC-11 cells was assessed in the presence of plasma derived from control, BTZ-treated or ATBL-treated mice at the indicated time points (*n* = 3/group). (D-E) Proliferation of RPMI and MPC-11 cells was assessed in the presence of plasma derived from control, BTZ-treated or ATBL-treated mice at the indicated time points (*n* = 3/group). (F) A schematic illustration of the macrophage extraction experimental design. BALB/c mice were induced by thioglycolate, and 24 h later intravenously injected with ATBL (1.95 ± 0.43 × 10^13^ liposomes/kg) BTZ-F (1 mg/kg) or vehicle control. Peritoneal macrophages were harvested 48 h later. (G) The percentage of M1 and M2 macrophages was evaluated by flow cytometry. Results are presented as mean ± SD. One-way ANOVA was used for the statistical analysis in B and C with multiple comparisons test. Two-way ANOVA was used for the statistical analysis in D-E and G with multiple comparisons test, adjusted p-value; *p < 0.05, **p < 0.01, ***p < 0.001, ****p < 0.0001. ns, not significant; MM, multiple myeloma; RPMI, RPMI8226; ATBL, AMD targeted bortezomib liposomes; BTZ-F, bortezomib free drug; Control, vehicle.

Our previous preclinical studies demonstrated that BTZ treatment induces MM cell aggressiveness via host cell activity rather than via direct effects on cancer cells^8,9^ Specifically, plasma from BTZ-treated mice induced MM cell migration and proliferation *in vitro.*(8) Furthermore, we found that BTZ treatment elevates the levels of M1 pro-inflammatory macrophages in the bone marrow where they secrete factors that support MM cell aggressiveness.^8,9^ Given these findings, we sought to determine whether ATBL induces pro-tumorigenic activities, similar to free BTZ. To this end, tumor-free BALB/c mice were treated with ATBL, BTZ-F or vehicle control. Blood samples were collected at 24, 72, and 168 h postdrug administration, and plasma was isolated for use in cell migration and proliferation assays (Figure 5A, for illustration). The migration assay demonstrated a significant increase in migratory properties of human RPMI and mouse MPC-11 MM cells in the presence of plasma from BTZ-F-treated mice compared to control, consistent with a previously published study.^8^ Interestingly, plasma from ATBL-treated mice did not induce significant changes in cell migration (Figure 5B,C). Similarly, plasma collected at later time points from ATBL-treated mice had no effect, ruling out the possibility of a delayed response to liposome-encapsulated BTZ. The proliferation assay demonstrated enhanced proliferation of RPMI and MCP-11 cells in the presence of plasma from BTZ-treated mice as shown by the significantly higher percentage of cells in the G2/M phase in the BTZ-treated group compared to control. This effect was absent in the presence of plasma from ATBL-treated mice at all the tested time points (Figure 5D,E).

Next, to investigate whether ATBL affects pro-tumorigenic host cells *in vivo*, the percentage of peritoneal M1 pro-inflammatory macrophages was assessed in mice treated with ATBL, BTZ-F or vehicle control (Figure 5F, for illustration). Unlike BTZ-F which elevated the level of M1 macrophages known to promote MM aggressiveness,^8,9^ ATBL had no effect (Figure 5G). Of note, neither ATBL nor BTZ-F affected M2- anti-inflammatory macrophages. Overall, these *in vitro* and *in vivo* experiments suggest that ATBL treatment does not induce host-mediated effects associated with MM aggressiveness, clearly differentiating it from BTZ treatment. The results can also explain the increased therapeutic effect of ATBL compared to BTZ.

### ATBL Is Effective against BTZ-Resistant Tumors

**Figure 6:**
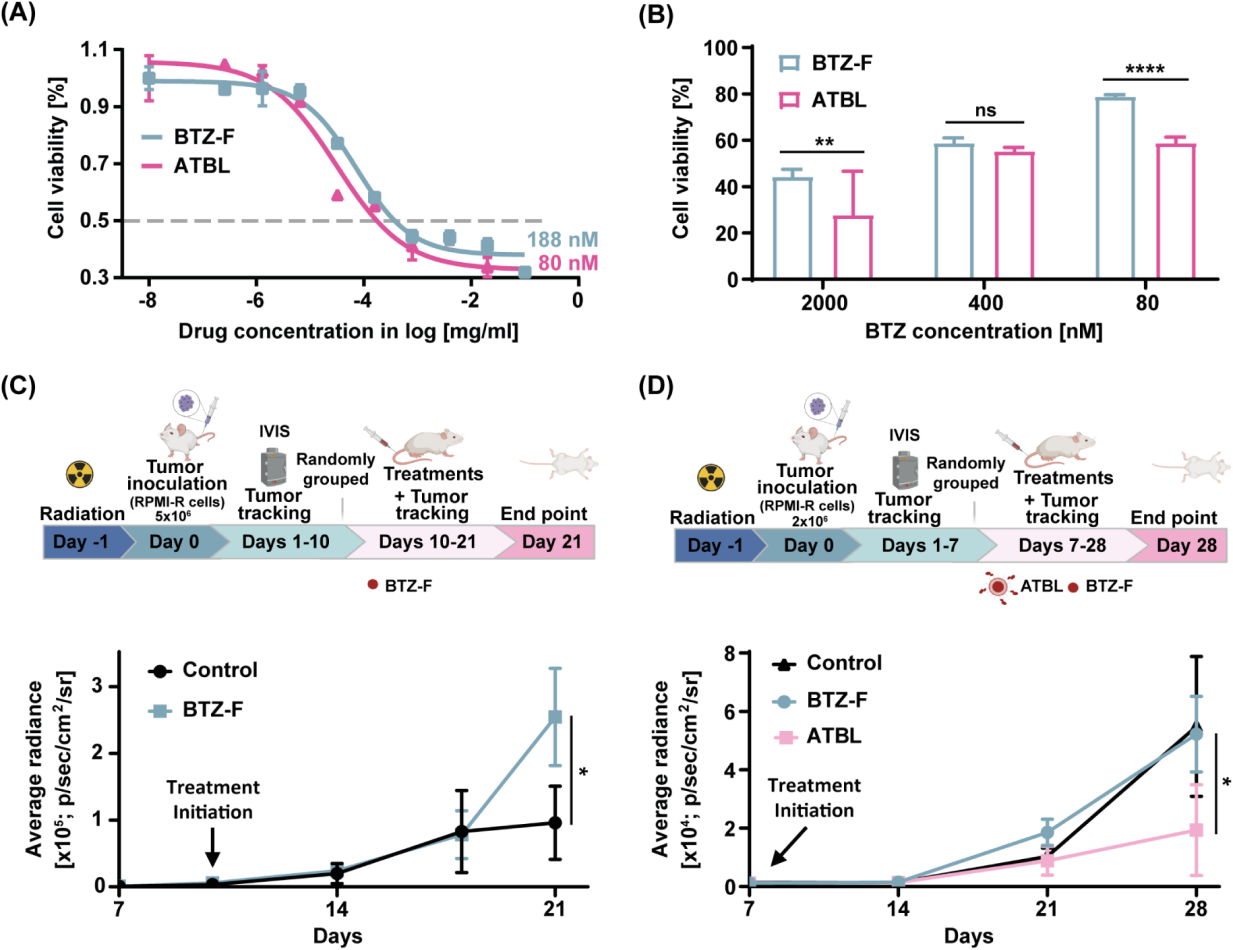
ATBL is effective against BTZ-resistant tumors. (A) The IC50 of BTZ and ATBL was determined in RPMI-R cells that display resistance to BTZ (*n* = 3/group). (B) RPMI-R cells were treated with ATBL or BTZ-F at the indicated concentrations for 24 h. Cell viability was assessed by flow cytometry (*n* = 4/group). (C) A schematic illustration of the experimental design: Eight-week-old SCID mice were systemically irradiated (250rad). After 24 h, MM RPMI-R cells were intravenously injected. When sufficient tumor burden was detected (day 10), BTZ-F (1 mg/kg) or control was intravenously administrated once a week. Tumor growth was monitored by IVIS (*n* = 4–5 mice/group). (D) SCID mice were treated as in C with adjustments. The number of inoculated RPMI-R cells was reduced to 2 × 10^6^, and treatment with BTZ-F (1 mg/kg), ATBL (1.95 ± 0.43 × 10^13^ liposomes/kg) or control was initiated on day 7 (*n* = 3–7 mice/group). Results are presented as mean ± SD. Two-way ANOVA was used for the statistical analysis in B-D with multiple comparisons test, adjusted p-value; *p < 0.05, **p < 0.01, ****p < 0.0001. ns, not significant; MM, multiple myeloma; IC50, half maximal inhibitory concentration; RPMI-R, RPMI8226 cells resistant to bortezomib; ATBL, AMD targeted bortezomib liposomes; BTZ-F, bortezomib free drug; Control, vehicle.

While we have shown that ATBL has better therapeutic activity than BTZ in its free form (Figure 3), this does not necessarily suggest that it is also effective against BTZ-resistant MM cells. To test this, we generated clones of RPMI cells adaptively resistant to high concentrations of BTZ *in vitro* (RPMI-R) as described in Materials and Methods and in Ghobrial et al.^37^ RPMI-R cells exhibited enhanced proliferation and migration when compared to parental RPMI (RPMI-P) cells, despite no change in CXCR4 expression (Figure S5). In RPMI-R cells, the IC50 of BTZ-F was 188 nM, higher than that of ATBL (80 nM) and considerably higher than that of BTZ-F in RPMI-P cells (11.9 nM). Importantly, the fold-change in IC50 of ATBL relative to BTZ-F was similar in RPMI-R and RPMI-P cells (Figures 6A,B and 2G).

To verify that the resistant and aggressive traits of RPMI-R cells observed *in vitro* were also evident *in vivo*, RPMI-R cells were inoculated in SCID mice, and tumor growth was monitored by IVIS. Due to the aggressive nature of the cells *in vivo* and *in vitro* (Figures 6C and S5), treatment was initiated on day 10 post tumor inoculation when tumors reached bioluminescence of approximately 2000–3000 average radiance [p/sec/cm2/sr]. This is in contrast to RPMI-P cells, for which treatment typically commenced after 21 days, as shown in Figure 3. Remarkably, after 21 days, RPMI-R cells were resistant throughout BTZ-F treatment (Figure 6C). In fact, mice treated with BTZ exhibited even higher tumor growth rates compared to control mice, probably due to BTZ-induced pro-tumorigenic host effects as previously published.^8,9^ Based on this *in vivo* study demonstrating aggressive and accelerated tumor growth rate, we reduced the number of inoculated cells and adjusted treatment schedules (as described in Materials and Methods). Under these conditions, ATBL achieved significant inhibition of tumor growth compared to BTZ-F even in this aggressive and resistant MM model (Figure 6D). These findings suggest that ATBL is effective against aggressive, BTZ-resistant MM.

## Discussion

Resistance to BTZ therapy remains an inevitable challenge encountered by all MM patients over time. BTZ resistance mechanisms are dependent on tumor-intrinsic and tumor-extrinsic factors.^7,38^ For example, recent studies have elucidated the involvement of mutations or upregulation of proteasome subunits, particularly β5, in conferring resistance to BTZ,^39,40^ while other studies have highlighted the pivotal role of host-mediated pro-tumorigenic responses.^8,9^ Specifically, our previous studies have shown that almost any type of anticancer drug can potentially trigger the host to upregulate various cytokines and growth factors in the plasma, attracting tumor-supporting cells to the tumor site. These effects promote tumor outgrowth, cancer cell aggressiveness, and tumor relapse.^41,42^ We previously demonstrated that BTZ elicits such a response by affecting macrophages, similar to chemotherapeutic agents. In particular, macrophages induced the expression of interleukin-1 beta (IL1β) and interleukin-16 (IL16), thereby contributing to MM aggressiveness.^8,9^ These pro-tumorigenic responses take place within hours following BTZ administration, likely attributed to its acute therapeutic dosage and relatively short half-life.^43^ Consequently, optimization of the duration of BTZ activity has emerged as a critical consideration for enhancing its efficacy. As such, substantial efforts have been directed toward the development of second and third-generation BTZ analogs aimed at ameliorating side effects while altering its half-life in order to increase therapeutic activity.^38^

Over the past two decades, studies have focused on novel drug delivery mechanisms utilizing nanoparticles to target specific anatomical sites including carcinogenic tissue.^44^ Among these, liposome nanocarriers, comprising a phospholipid bilayer structure capable of encapsulating drugs within their internal compartment, have gained attention.^45^ Notably, the first liposomal formulation approved by the FDA, Doxil, encapsulates doxorubicin and is utilized for the treatment of diverse malignancies.^46^ Despite the potential of liposomes as a cancer treatment strategy, relatively few liposome-based drugs have been clinically approved, emphasizing the challenges of translating preclinical findings into clinical scenarios. The advantage of liposomes lies in their potential for targeted drug delivery facilitated by the enhanced permeability and retention (EPR) effect, coupled with prolonged pharmacological activity resulting from sustained drug release within target tissues. These properties mitigate adverse effects while augmenting drug concentrations.^45^ In light of the limitations associated with BTZ therapy, we sought to develop liposomal nanocarriers encapsulating BTZ that can target the bone marrow. The encapsulation of BTZ within liposomes was previously demonstrated.^47^ We have taken a parallel approach, where BTZ was effectively loaded using mannitol and acetic acid gradients. To generate the targeting moiety, we developed targeted liposomes incorporating a CXCR4 antagonist, AMD, to the BTZ liposomes. CXCR4, primarily expressed by immune cells and in the bone-marrow, plays a central role in retaining marrow cells within the bone matrix through its interaction with its chemokine ligand, SDF-1.^48^ Disruption of the SDF-1-CXCR4 axis has been shown to induce the release of bone-marrow cells into the circulation and sensitizes CXCR4-expressing MM cells to therapy.^49^ Our study demonstrates that ATBL exhibits significantly enhanced therapeutic activity compared to other forms of BTZ therapies, due in part to the expression of the CXCR4 as a target moiety. Importantly, these results were found to be valid both *in vitro* and *in vivo*, indicating the potential therapeutic value of ATBL for MM. Furthermore, in addition to the targeting effect of AMD, it is also plausible that AMD-induced disruption of MM cell retention in the bone niche sensitized the MM cells to BTZ therapy, as previously reported.^50^ Indeed, such studies demonstrated the additive advantage of combining AMD with BTZ when both drugs are administered as free drugs. Importantly, our study highlights a clinical advantage given that fewer administrations are necessary with ATBL compared to BTZ in its free form. We showed that a weekly ATBL administration was sufficient to achieve substantial therapeutic activity, thus potentially reducing the number of patient visits to the clinic. Overall, AMD may have a dual effect in our system, since it serves both as a targeting moiety and also as a MM sensitizing agent. It should be noted that in the clinical setting, AMD is given at a dose of 240 μg/kg^51^ whereas BTZ is given at a dose of 1.3 mg/m2.^52^ The desired ratio between the two drugs can be achieved when generating ATBL; yet, further investigation is required to identify the optimal combined concentrations of AMD and BTZ within ATBL liposomes for clinical use.

Our study demonstrates that ATBL has superior therapeutic efficacy compared to both BTZ-F and BTZ-L. Furthermore, we demonstrate its potential utility for managing BTZ-resistant and aggressive MM tumors. In an *in vitro* model of BTZ-resistant MM, we demonstrated that while the IC50 of BTZ-F was 188 nM, the IC50 of ATBL was 3-fold lower (∼80 nM), suggesting greater efficacy of ATBL even in the context of resistant tumors. These findings were consistent with the *in vivo* results demonstrating improved therapeutic outcomes with ATBL compared to BTZ-F in this resistant model. Although the precise mechanism underlying the enhanced sensitivity of BTZ-resistant MM cells to ATBL remains elusive, we speculate that ATBL achieves better results as it does not induce tumor-supporting, host-mediated effects shown to occur in response to BTZ in its free form.^8^ Indeed, we found that ATBL had no effect on MM cell proliferation and migration. In addition, the levels of M1-pro-inflammatory macrophages, which have been shown to support MM aggressiveness, were similar when using ATBL or BTZ-F in in vitro and ex vivo experimental settings. It will be of interest, however, to examine directly whether the superior therapeutic effect of ATBL observed in vivo is also due to reduced host pro-tumorigenic effects by means of decreased percentage of M1 macrophages, as shown in previous studies.^8,9^ Notably, MM-resistant cells displayed accelerated tumor growth *in vivo* in response to BTZ treatment compared to controls, demonstrating that the pro-tumorigenic host-mediated effects associated with BTZ therapy are indeed valid in our experimental setting. Importantly, we also found that the therapeutic activity of ATBL is associated with the expression of CXCR4 in MM cells. Our *in vivo* experiment substantiates that diminished CXCR4 expression in MM cells correlates with reduced efficacy of ATBL treatment. These findings are consistent with prior studies highlighting the pivotal role of CXCR4 expression in MM cells.^19^ Taken together, our study reveals that MM cells lacking CXCR4 expression fail to elicit comparable therapeutic responses to those expressing CXCR4. This highlights the potential utility of CXCR4 as a companion diagnostic biomarker for ATBL therapy.

## Conclusions

Our study shows that AMD-L as delivery vehicles mitigate adverse effects while enhancing therapeutic efficacy within bone tissues. The encapsulation of BTZ in liposomes containing a bone targeting moiety in the form of AMD represents a promising therapeutic strategy for MM, and possibly other bone-related diseases.

## Methods

### Liposome Preparation

#### AMD-Targeted Liposomes (AMD-L)

Liposomes were prepared using the ethanol injection method. The following lipids were used: hydrogenated soybean phosphatidylcholine (HSPC) (Lipoid, Ludwigshafen, Germany, Cat# 525600); cholesterol (Sigma-Aldrich, Rehovot, Israel, Cat# C8667); 1,2-distearoyl-*sn*-glycero-3-phosphoethanolamine-N-[carboxy(polyethylene glycol)-1000] (sodium salt) (DSPE-PEG1000-COOH) (Biopharma PEG, Watertown, MA, USA Cat# LP096017). Lipids were combined at a molar ratio of 55:40:5 (HSPC:cholesterol:DSPE-PEG1000-COOH), dissolved in absolute ethanol and warmed (65̊C). Then, the lipid suspension was injected into an aqueous solution of acetic acid (150 mM) and mannitol (150 mM) adjusted to pH = 9 to form multilamellar liposomes.^26^ The liposome mixture was then downsized by extrusion through 400-, 200-, 100-, and 80- nm pore-size polycarbonate membranes (LIFEGENE, Israel, Cat# 10417106, 110606, 110605, and 110604, respectively) using a LIPEX extruder (Northern Lipids, Canada) at 65 °C, with a maximum working pressure of 15 bar to obtain homogeneous 100 nm liposomes. Liposomes were dialyzed against 50 mM MBS pH = 6 (1:1000 volume ratio) using a 12 to 14 kDa dialysis membrane (Spectrum Laboratories Inc., USA Cat# 110000651) at 4 °C and exchanged three times (after 1 h, 4 h, and overnight).

AMD was conjugated to the phospholipid backbone of the liposome surface via a nucleophilic substitution carbodiimide cross-linking reaction. First, 12 mg/mL of *N*-(3- dimethylaminopropyl)-N’-ethyl carbodiimide hydrochloride (EDC) (Sigma-Aldrich, Cat# 03450) and 5.5 mg/mL of *N*-hydroxysulfosuccinimide sodium salt (Sulfo-NHS) (Chem Scene, Monmouth Junction, NJ, USA, Cat# CS-W002213) in PBS pH = 7 were added to the liposome mixture and mixed using an orbital shaker (700 rpm) at 25 °C for 30 min.^27^ Next, the liposomes were dialyzed against PBS pH = 7 in the same manner as described above. Second, 10 mg/mL AMD (ApexBio Technology, Houston, TX, USA, Cat# A2025) in PBS pH = 7 was added and mixed (600 rpm) at 25 °C for 2 h to form an amide linkage. Next, the liposomes were dialyzed against PBS pH = 6, to remove excess AMD. For validation of the conjugation process we used thin layer chromatography (TLC). Silica gel plates (Merck, Germany Cat# 105554) served as the stationary phase and chloroform: methanol: ammonia hydroxide 25% in H_2_O at a ratio of 5:12.5:3 served as the mobile phase.^53^

#### Cy5- or Rhodamine-Labeled Liposomes

Cy5- (ex. 651 nm/em. 670 nm) or rhodamine-labeled (ex. 546 nm/em. 567 nm) liposomes were prepared using the ethanol injection method, as described above. For Cy5 labeled liposomes, 1,2-distearoyl-*sn*-glycero3-phosphoethanolamine (DSPE) (Lipoid, Germany, Cat# 565400) was mixed with Cy5 se(mono so3) (BLD pharm, China Cat# BD759435), in a molar proportion 1:1 (DSPE:Cy5) to yield Cy5-DSPE synthesized lipids. For the rhodamine labeled liposomes (Rhod-EMPTY-L) we used 16:0 Liss Rhod PE (Sigma-Aldrich, Rehovot, Israel, Cat# 810158P). Subsequently, the labeled lipid was added to the lipid mixture at a 0.4% molar ratio, achieving labeled liposome compositions of HSPC: cholesterol: DSPE-PEG1000-COOH: DSPE-Cy5 or Liss Rhod PE in molar percentages of 54.6:40:5:0.4. The liposome mixture was extruded and AMD was conjugated to the Rhod-EMPTY-L, as described above, to reach a final concentration of 50 mM total lipids. Furthermore, the stability of rhodamine AMD liposomes (Rhod-AMD-L) was characterized by determining mean size diameter (nm) and poly dispersity index (PDI) using the Zetasizer Ultra system (Figure S6A,B). Fluorescence intensity of 16:0 Liss Rhod PE was measured at 500 nm using a plate reader (Tecan, Mannedorf, Switzerland), after samples were dialyzed at 4 and 37 °C, and at 4 time points over 72 h (Figure S6C,D). Labeled liposomes were used within 24 h after their preparation.

#### BTZ Liposomes (BTZ-L)

Active loading of BTZ into liposomes was performed using a concentration gradient of mannitol and acetic acid, where the concentration of mannitol and acetic acid was higher inside the liposomal core compared to the external medium.^28,29^ In addition, we created a transmembrane pH gradient between the exterior (pH = 6.5) and the interior (pH = 9) environments of the liposomes.^30^ BTZ (Selleckchem, Houston, TX, USA, Cat# S1013) was dissolved in 10% (w/w) DMSO and added to the liposomes to create bortezomib liposomes (BTZ-L) or to AMD-L to create ATBL. BTZ was added to reach a final concentration of 2, 2.5, or 3 mg/mL, as indicated in the text. The mixture was incubated overnight at 25 °C with mixing at 600 rpm. Subsequently, the liposomes were dialyzed against PBS pH = 7 in the same manner as described above.

### Liposome Characterization

#### Physical Characterization

The physical characteristics of liposomes, including mean size diameter (nm), particle size distribution, poly dispersity index, and particle concentration (particles/ml) were measured in PBS. Zeta potential (mV) was measured using dynamic light scattering with a Zetasizer Ultra (ZSP, Malvern, UK).

#### Quantification of Encapsulated BTZ Concentration

For the quantification of BTZ, a 325 nm wavelength absorbance calibration curve was constructed using a plate reader. Subsequently, liposomes were disintegrated using methanol in a ratio of 1:1. The concentration of the loaded drug was measured, and calculated using a calibration curve. The encapsulation efficiency (EE%) was determined as the percentage of the final loaded BTZ concentration. The average concentration of ATBL and BTZ-L was 0.965 ± 0.224 mg/mL. Bortezomib encapsulated units per liposome was calculated using the bortezomib concentration and the liposome particle concentration as follows:
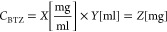
1
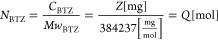
2
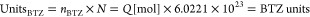
3
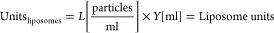
4
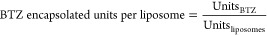
5

#### Quantification of Conjugated AMD to Liposome Surface

For the quantification of AMD we used ninhydrin reaction that forms a luminous coordination complex with the secondary amine on the AMD molecule. First, an AMD calibration curve was prepared. Each tube of the calibration curve contained AMD, dissolved in PBS, at a known concentration, and Triton-X100 (1%). In addition, the liposome samples (25 mM, lipid concentration) were disintegrated using Triton-X100 (1%), and shaken at 650 rpm for 1 h at 80 °C. Subsequently, the samples were centrifuged (13,000 rpm, 10 min, 4 °C) to obtain a clear supernatant. In parallel, a ninhydrin solution was mixed with citrate buffer in 1:1 ratio, and prepared as described in.^54^ Next, the samples (calibration curve and the clear supernatant from liposomes) were added to the mixed solution in a ratio of 1:2 and incubated at 100 °C for 15 min. The samples were then transferred into a 96-well plate and absorbance was measured at 570 nm using a plate reader. BTZ-L and EMPTY-L were used as blanks. A linear calibration curve for AMD was obtained using the absorbance values to quantify the concentration of AMD (mg/mL) on the liposome surface. The average concentration of AMD on ATBL was 0.41 ± 0.1 mg/mL. Then, the number of AMD units per liposome surface was calculated using the AMD concentration and the liposome particle concentration as follows:
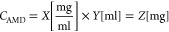
6
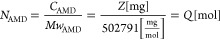
7
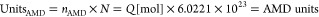
8
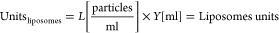
9
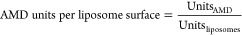
10

#### Cryogenic Transmission Electron Microscopy (Cryo-TEM)

Cryo-TEM imaging was conducted at the Technion Center for Electron Microscopy of Soft Matter, utilizing a FEI Talos 200C high-resolution TEM from Thermo Fisher Scientific. The specimen preparation took place in a controlled environment vitrification system under conditions of 25 °C and 100% relative humidity. A diluted ATBL solution (1 mM, lipid concentration) was applied to a carbon-coated perforated polymer film on a 200-mesh TEM grid held by tweezers. The solution was spread into a thin film using a filter paper-covered metal strip, and subsequently rapidly frozen by immersing it in liquid ethane at −183 °C. Next, the grid was transferred to a Gatan 626 cryo-holder and imaged at −175 °C. The images were digitally captured using an FEI Falcon III digital camera, with image contrast enhanced by a Volta ″phase plate″

#### BTZ Release Profile

Using a 12- to 14-kDa dialysis membrane, ATBL and BTZ-F were dialyzed against 50% fetal bovine serum (FBS, Biological Industries, Israel, Cat# 10270–106) and 50% PBS pH 7 (1:10 volume ratio) with mixing (200 rpm) at 37 °C. The level of free BTZ in the extra liposomal buffer was quantified at the desired time intervals using the plate reader. The sample was then returned to incubation until the next measurement. The percentage of BTZ release was determined by the ratio of free BTZ to total encapsulated BTZ concentration.

#### Stability Characterization of ATBL

The mean size diameter (nm), particle size distribution, poly dispersity index, and zeta potential (mV), of ATBL stored at 4 °C were measured at five time points over 12 weeks using a Zetasizer Ultra instrument.

The concentration of AMD and BTZ on the different liposomes are summarized in Table S1.

### Cell Culture

CAG, U266 and RPMI8226 (RPMI) human MM cell lines, (American Type Culture Collection; ATCC); KMS-11 (originally from the Japanese Collection of Research Bioresource cell bank) human MM cell line, and MPC-11 murine myeloma cell line (kindly provided by Professor Ralph Sanderson, University of Alabama, USA) were used. Cells were routinely tested to be mycoplasma-free. Cells were cultured in RPMI-1640 medium (Sigma-Aldrich, Rehovot, Israel, Cat# R8758) supplemented with 10% fetal bovine serum (FBS, Biological Industries, Israel, Cat# 10270–106), 1% l-glutamine (Biological Industries, Israel, Cat# 03–020–1B), 1% sodium pyruvate (Sigma-Aldrich, Rehovot, Israel, Cat# S8636) and 1% pen-strep-neomycin in solution (Biological Industries, Israel, Cat# 03–034–1B). All cells were cultured at 37 °C in 5% CO_2_ for no more than 6 months after being thawed from the authentic stocks.

### The Generation of Resistant MM Clones

MM RPMI8226 cells resistant to BTZ (RPMI-R) were generated as previously described.^37^ Briefly, RPMI8226 cells were grown in RPMI medium supplemented with 15% FCS, in the presence of a gradually increasing concentration of BTZ (0.5–200 nM). Both medium and drug were refreshed every 72 h, and following each increment in concentration, cells were grown for at least 4 weeks, before proceeding to the next higher concentration.

### Flow Cytometry Analysis

The percentage of CXCR4 expression on several MM cell lines was evaluated in both *in vitro* and *in vivo* experiments. The cells were immunostained with CD184, (Biolegend, San Diego, CA, USA, Cat# 306527). In some experiments, liposome uptake was assessed using Cy5 labeled liposomes or rhodamine labeled liposomes in both *in vitro* and *in vivo* experiments. 7AAD and Annexin V were used to assess cell apoptosis as previously described.^55^ For cell cycle analysis, MM cells were stained with propidium iodide (PI) (Biolegend, San Diego, CA, USA, Cat# 421301 or Cat# 79997) for the evaluation of DNA content as previously described.^56^ In other experiments, the frequency of M1 and M2 macrophages was assessed using the following antibodies: M1 pro-inflammatory macrophages were defined as CD11b+/F4/80+/CD206-/CD11c+; M2 anti-inflammatory macrophages were defined as CD11b+/F4/80+/CD206+/CD11c-. At least 100,000 events were acquired using the LSRFortessa flow cytometer (BD) and analyzed with FlowJo 10.

### SDF-1 Measurement

MM cells (1 × 10^6^/ml) were cultured for 24 h in serum-free medium in the presence of escalating doses of AMD as indicated in the figure, to generate conditioned medium (CM). SDF-1 levels in CM were determined by specific ELISA (Biotest, Minneapolis, MN, USA Cat# DY460) according to the manufacturer’s instructions.

### Transwell Migration Assay

MM cell chemotaxis or migration was quantified using the Boyden chamber assay as previously described.^8^ Briefly, MM cells were incubated with 1% FBS and were cultured with the different liposomes for 90 min or with serum-free medium. Subsequently, the MM cells (0.5 × 10^6^, for SDF-1 study, and 1 × 10^6^ for the migration study) were placed in the upper compartment of the chamber (0.8 μm insert, Corning, NY, USA Cat# FAL353097) coated with fibronectin. The lower compartment was filled with 10% or 1% FCS RPMI medium containing 30 nM SDF-1 or 5% plasma collected at different time points from mice treated with ATBL or BTZ-F. After 24 h, cells that migrated to the bottom compartment were collected and counted using cell counting chamber or flow cytometry using 7.3 μm counting beads (Bangs Laboratories, Fishers, IN, USA, Cat#FSDG007), according to the manufacturer’s instructions.

### Liposomal Cellular Uptake

MM cells (0.5 × 10^6^/ml) were seeded and incubated for 30, 60, and 90 min with freshly prepared Cy5- or rhodamine labeled AMD-L or EMPTY-L at the concentrations described in the text. Subsequently, the culture medium was removed, and the cells were rinsed with PBS and resuspended in fresh medium. Cellular uptake of liposomes was measured using flow cytometry.

### PrestoBlue Viability Assay

MM cells were seeded in a 96-well plate (25,000 cells/well) in RPMI medium and cultured for 24 h in the presence of different liposomes at escalating concentration, as indicated in the figures and text. Subsequently, PrestoBlue Cell Viability Reagent (ThermoFisher, Waltham, MA, USA Cat# A13261) was added)10 μL of reagent for 90 μL media) and incubated for 80 min. The absorbance was measured at a wavelength of 560 nm Excitation, 590 nm Emission using a plate reader.

### Cell Doubling Time and Growth Rate

MM cells were seeded in a 24-well plate (500,000 cells/well) in full medium. After 72 h, the cells were collected and counted using Vi-CELL XR cell viability analyzer (Beckman Coulter). The growth rate and doubling time were calculated using omni calculator (omnicalculator.com) with the following equations:
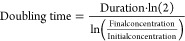
11
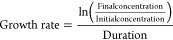
12

### CXCR4 Knockdown

A single guide RNA (gRNA) for CXCR4 (forward: 5′CACCG AGGGGACTATGACTCCATGA 3′; reverse: 5′AAAC TCATGGAGTCATAGTCCCCT C 3′) was cloned into the lentiCRISPR v2 vector plasmid (Addgene, Watertown, MA, USA Cat# 52961) containing puromycin resistance using the Golden Gate assembly reaction as described.^57^ Next, lentiviral particles were generated by cotransfecting HEK-293FT cells with packaging (δNRF) and envelope (VSV-G) plasmids together with lentiCRISPR v2 vector containing gRNA specific for CXCR4 gene in Dulbecco’s modified Eagle’s medium (DMEM, Sigma-Aldrich, Rehovot, Israel Cat# 140005680). After 24 h, fresh RPMI media was added, and 2 days later, supernatants containing viral particles were centrifuged at 3000 rpm for 10 min and filtered through a 0.45 μm syringe filter. The viral particles were then used to infect MM cells that were cultured for 72 h before selection with puromycin antibiotic (Sigma-Aldrich, Rehovot, Israel, Cat# P8833) added twice a week for 3 weeks. Flow cytometry was used to validate CXCR4 knockdown expression. In parallel, we used RPMI-WT* cells as a control. These cells underwent the same genetic manipulation as the RPMI-KD cells, but without the inclusion of the guide RNA.

### Expression of Luciferase in RPMI8226 Cells

MM cells were infected with the pGreenFire1-CMV Positive Control Lentivector plasmid (Kindly provided by Professor Yotam Bar-on, Technion, Israel) expressing GFP and firefly luciferase. Next, lentiviral particles were generated by cotransfecting HEK-293FT cells, in a similar procedure described above. The viral particles were then transduced into the RPMI cells. Subsequently, cells stably expressing luciferase were selected using cell sorting for GFP^+^ cells using FACSAria IIIu (BD). Luciferase expression was tested using an IVIS Lumina X5 (PerkinElmer, Waltham, MA, USA).

### Animal Tumor Models and Treatments

The use of animals and experimental protocols were approved by the Animal Care and Use Committee of the Technion. Female BALB/c and combined immunodeficient (SCID) female mice (8 weeks of age) were purchased from Envigo, Israel. All mice were maintained under specific pathogen-free conditions in the animal facility. The SCID mice underwent whole-body radiation at a total dose of 250 rad (X-RAD320, Precision, Wisconsin, US). After 24 h, the mice were intravenously injected with MM cells as indicated in the text (5 × 10^6^/mouse for RPMI cells and 2 × 10^6^/mouse for RPMI-R cells). After 1–4 weeks, when sufficient tumor growth was detected by bioluminescence IVIS Lumina X5 (PerkinElmer, Waltham, MA, USA), mice were randomly grouped and different treatments were initiated using EMPTY-L, BTZ-F, BTZ-L, AMD-L, ATBL. In all relevant liposomes, BTZ was administered at a final dose of ∼ 1 mg/kg, and AMD at a final concentration of ∼ 0.5 mg/kg (see Table S1). Liposomes were administeredat a final dose of 1.89 ± 0.43 × 10^13^ liposomes/kg, as indicated in the text. All treatments were intravenously injected once a week. Tumor volume was measured once a week using IVIS imaging. In some experiments, ATBL was given at a final BTZ concentration of 0.5, 2, or 5 mg/kg, as indicated in the text. The number of mice per group was set between 4 and 6/group. Mice were followed up daily and when mice were showing signs of paralysis or weight loss, they were sacrificed.

### Plasma Sample Collection

Blood obtained from mice at different time points after treatment administration was collected into EDTA tubes by submandibular vein puncture. Subsequently, plasma was isolated by centrifugation of whole blood at 4 °C, 1000 × *g* for 20 min. The plasma was stored in aliquots at – 80 °C until further use.

### Macrophage Extraction

BALB/c mice were intraperitoneally injected with 3 mL of 4% thioglycolate, and 24 h later, were injected intravenously with ATBL, BTZ-F or vehicle control. After an additional 48 h, macrophages were collected by peritoneal lavage as previously described.^8,58^ Macrophage frequency was measured using flow cytometry as described above.

### In Vivo Animal Imaging

Bioluminescent imaging of luciferase-expressing tumor cells was performed Lumina X5 (PerkinElmer, Waltham, MA, USA), as previously described.^8^ Briefly, mice were injected intraperitoneally with d-luciferin (150 mg/kg) and were anaesthetized using isoflurane. Subsequently, the mice were placed onto the IVIS stage, with continuous exposure to isoflurane and oxygen for the maintenance of anesthesia. Bioluminescence emitted from the MM cells was detected by the IVIS camera system. Images were acquired for assessing tumor burden using a log-scale threshold set at 3 × 10^4^–2.5 × 10^7^. Measurements of the average radiance (photon/s/cm^2^/square radiance) were calculated. Image quantification was performed using Living Image software v. 4.7.2 (PerkinElmer).

### Biodistribution and Quantification of Liposome Delivery into Tumors

Rhodamine-labeled liposomes loaded with gadolinium (Gd) were prepared using the ethanol injection method as described above. Briefly, 16:0 Liss Rhod PE was added to the lipid mixture at 0.4% molar ratio in the same molar ratios as mentioned above, which was then injected into PBS solution containing diethylenetriaminepentaacetic acid Gd (III) (167 mg/mL) dihydrogen salt hydrate (SigmaAldrich, Rehovot, Israel, Cat# 381667). The liposome mixture was extruded and AMD was conjugated to the Gd liposome (Gd-AMD-L) surface, as described above, to reach a final concentration of 50 mM total lipids. Furthermore, stability of the liposomes was characterized by determining mean size diameter (nm), particle size distribution, and poly dispersity index using a Zetasizer Ultra system (Figure S6A,B). Gd release from liposomes was measured using inductively coupled plasma optical emission spectroscopy (ICP-OES, PlasmaQuant PQ 9000 Elite; analytik jena, Jena, Germany) after the samples were dialyzed at 4 and 37 °C and at 4 time points over 72 h (Figure S6E). Subsequently, 100 μL of the Gd-AMD-L (8.10 × 10^12^ liposomes/ml) was intravenously injected to SCID mice bearing MM RPMI tumors, 21 days after tumor inoculation. Mice were sacrificed at different time points (4, 12, 24, and 48 h), as indicated in the text, and organs including brain, liver, lungs, bones, and kidney, as well as blood were collected. All the tissues were weighed and burned at 550 °C for 5 h, and their ashes were dissolved in 1% nitric acid. The samples were analyzed using ICP-OES where Gd emission was measured at 303.284 nm. Gd concentrations were calculated by a calibration curve that was prepared using Gd ICP standard (AccuStandard, New Haven, USA, Cat#ICP-19N-1). The values were calculated per the dose injected and were further normalized to the organ’s weight. In some experiments, to study biodistribution of the drug in the bone-marrow, the bones were collected, and femurs and tibiae were flushed with sterile PBS to obtain bone-marrow. Subsequently, bone-marrow cells were filtered through a 70 μm pore size cell strainer (BD Biosciences, Bedford, MA, USA, Cat#258368). Red blood cells (RBC) were lysed using a lysis buffer (8.26 g/L ammonium chloride, 1 g/L sodium bicarbonate and 0.01 M EDTA). Cellular uptake of liposomes was detected using flow cytometry.

### Tissue Preparation and Immunostaining

Bone samples were decalcified (National Diagnostics, Atlanta, GA, USA) and subsequently formalin-fixed, embedded in paraffin and sectioned at 20 μm thickness, as previously described.^8^ Slides were scanned using Panoramic 250 Flash III scanner (3DHISTECH, Budapest, Hungary). For the toxicity study, organs were placed in 4% paraformaldehyde pH = 7.2, and subsequently stained with H&E. The tissue samples were assessed by an external pathologist in a blinded manner (Path-Logica Ltd. Rehovot Israel).

### Statistical Analysis

For adequate statistical power, all experiments were performed with at least three biological repeats and two technical repeats. Data are expressed as mean ± SE or SD, as indicated in the figure legend. The statistical significance for the *in vitro* experiments was determined by either two-tailed Student *t* test for a comparison between two groups, or one or two-way ANOVA for a comparison between multiple groups, followed by Tukey posthoc statistical test, using GraphPad prism 10.0 software (GraphPad Software, Inc., La Jolla, CA, USA). For *in vivo* studies, *n* = 3–6 mice/group were used unless indicated otherwise. All mice were randomly grouped before treatment was initiated. Animals were excluded from the analysis if mice died during the experiment or demonstrated pathological conditions that are not related to their disease. Differences between all groups were compared with each other, and statistical significance was set at *p* < 0.05, and designated as follows: *, *p* < 0.05; **, *p* < 0.01; ***, *p* < 0.001, ****, *p* < 0.0001 with a 95% confidence interval.

## Supporting Information Available

Drug concentrations in the different liposome types (Table S1); construction and characterization of ATBL (Figure S1); levels of CXCR4, and its ligand SDF-1 on different MM cells, ATBL uptake, and MM cell apoptosis (Figure S2); generation and characterization of RPMI CXCR4 knockdown cells (Figure S3); ATBL biodistribution and toxicity (Figure S4); characterization of RPMI cells resistant to BTZ therapy (Figure S5); stability characterization of Rhod-AMD-L and Gd-AMD-L (Figure S6) (PDF)
