# Supplementary material for: Bone Marrow-Targeted Liposomes Loaded with Bortezomib Overcome Multiple Myeloma Resistance
Source: ACS Nano. 2025 Mar 21;19(12):11684–701. doi: 10.1021/acsnano.4c10597 (PMC11966756; doi:10.1021/acsnano.4c10597)
Supplement: Supplementary file 1 — nn4c10597_si_001.pdf [file nn4c10597_si_001.pdf]

Supporting Information

Bone marrow-targeted liposomes loaded with bortezomib overcome multiple myeloma resistance

Rotem Menachem[1,2,3], Igor Nudelman[2,3], Avital Vorontsova[1,2], Ido Livneh[1,2], Mor Sela[2,3], Madeleine Benguigui[1,2], Bar Manobla[1,2], Yael Shammai[2,3], Abhilash Deo[1,2], Chen Buxbaum[1,2], Ron Bessler[4], Ziv Raviv[1,2], Jeny Shklover[2,3], Josué Sznitman[4], Aaron Ciechanover[1,2], Avi Schroeder[2,3], Yuval Shaked[1,2]

[1] Department of Cell Biology and Cancer Science, Rappaport Faculty of Medicine, Technion–Israel Institute of Technology, Haifa, 3525422, Israel

[2] Rappaport Technion Integrated Cancer Center, Technion – Israel Institute of Technology, Haifa, 3525422, Israel.

[3] Faculty of Chemical Engineering, Technion – Israel Institute of Technology, Haifa, 3200003, Israel.

[4] Faculty of Biomedical Engineering, Technion–Israel Institute of Technology, Haifa, 3200001, Israel

Table S1

Liposome	Abbreviation	AMD concentration [mg/ml]	AMD molecules/ liposome [x10^4]	BTZ concentration [mg/ml]	BTZ molecules/ liposome [x10^5]
Empty non-targeted liposome	EMPTY-L	0	0	0	0
AMD liposome	AMD-L	0.334±0.047	7.079±0.592	0	0
Bortezomib liposome	BTZ-L	0	0	1.009±0.279	0.963±0.298
AMD targeted bortezomib liposome	ATBL	0.413±0.106	9.916±1.461	0.920±0.170	1.223±0.226

Table S1: Drug concentrations in the different liposome types. The table lists the different drugs included per liposome type. The drug concentration within the liposomes and the number of drug molecules per liposome are indicated.

Figure S1

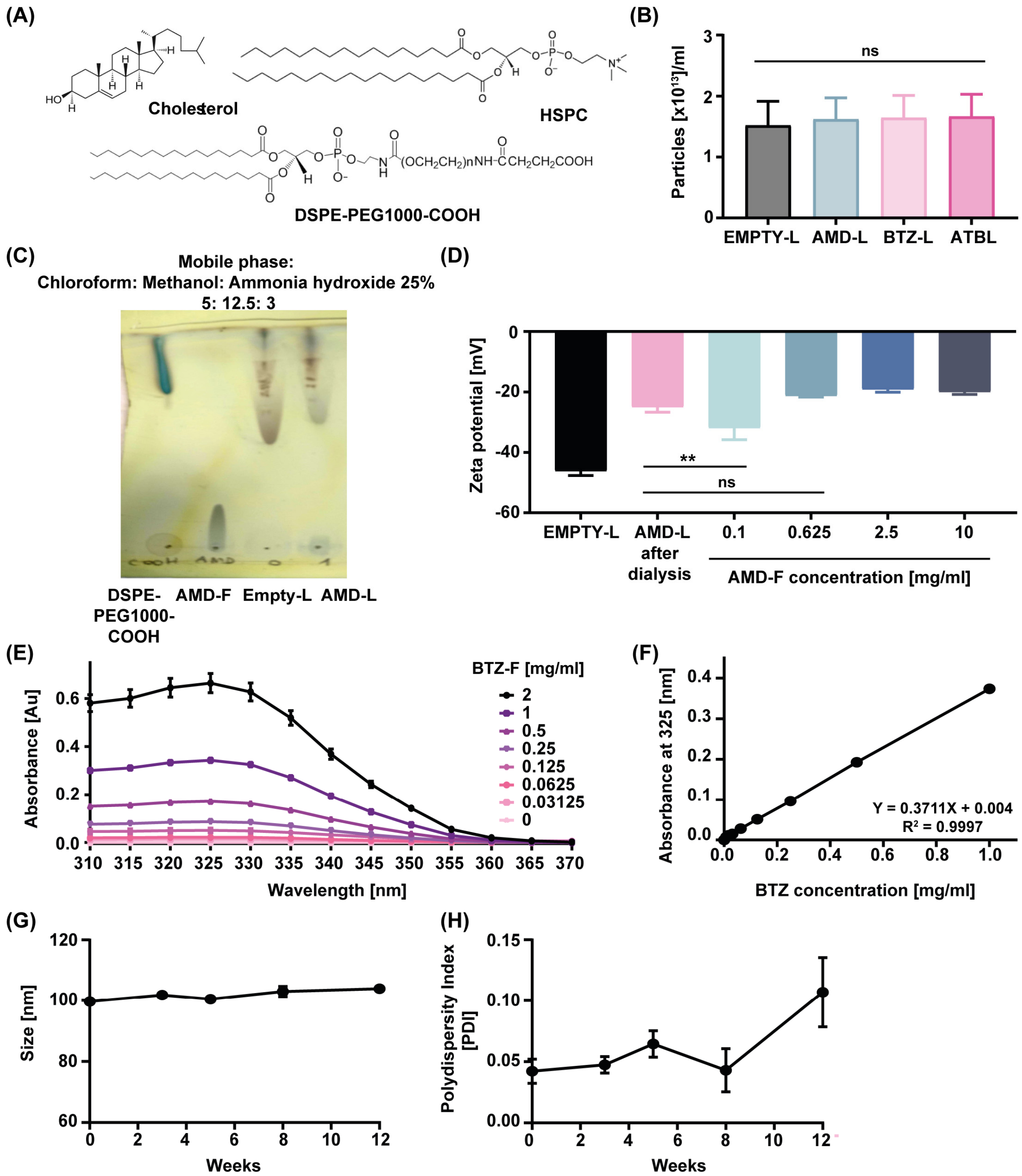
(A)
Cholesterol
HSPC
DSPE-PEG1000-COOH
(B)
Particles [x10^13]/ml
ns
EMPTY-L AMD-L BTZ-L ATBL
(C)
Mobile phase:
Chloroform: Methanol: Ammonia hydroxide 25%
5: 12.5: 3
DSPE-PEG1000-COOH AMD-F Empty-L AMD-L
(D)
Zeta potential [mV]
**
ns
EMPTY-L AMD-L after dialysis 0.1 0.625 2.5 10
AMD-F concentration [mg/ml]
(E)
Absorbance [Au]
BTZ-F [mg/ml]
2
1
0.5
0.25
0.125
0.0625
0.03125
0
Wavelength [nm]
(F)
Absorbance at 325 [nm]
Y = 0.3711X + 0.004
R² = 0.9997
BTZ concentration [mg/ml]
(G)
Size [nm]
Weeks
(H)
Polydispersity Index [PDI]
Weeks

Figure S1: Construction and characterization of ATBL. (A) Chemical structures of HSPC, cholesterol and DSPE-PEG1000-COOH which make up the liposomes. (B) Liposome particle concentrations in the different treatment groups (n = 10-13/group) (C) The conjugation of AMD to liposome surface was assessed using Thin-layer chromatography (TLC). (D) Zeta potential (mV) measured by Zetasizer (n=3/group). (E) Quantification of the encapsulated BTZ concentration using an absorbance scan measured over a range of wavelengths (n=3/group). (F) A calibration curve of known BTZ concentrations at 325 nm wavelength (n=3/group). (G-H) Liposome mean diameter (nm) (n=3/group) (G), and Polydispersity index (PDI) (n=3/group) (H) measured by Zetasizer over 12 weeks are presented. Results are presented as mean ± SD. One-way ANOVA was used for the statistical analysis of B and D. Statistical significance is shown only between ATBL after dialysis to 0.1 or 0.625 of AMD-F, adjusted p-value; **$p<0.01$. ns, not significant; ATBL, AMD targeted bortezomib liposomes; BTZ, bortezomib; BTZ-L, bortezomib liposomes; AMD, AMD3100; AMD-F, AMD3100 free drug; AMD-L, AMD3100 liposomes; EMPTY-L, empty non-targeted liposome.

Figure S2

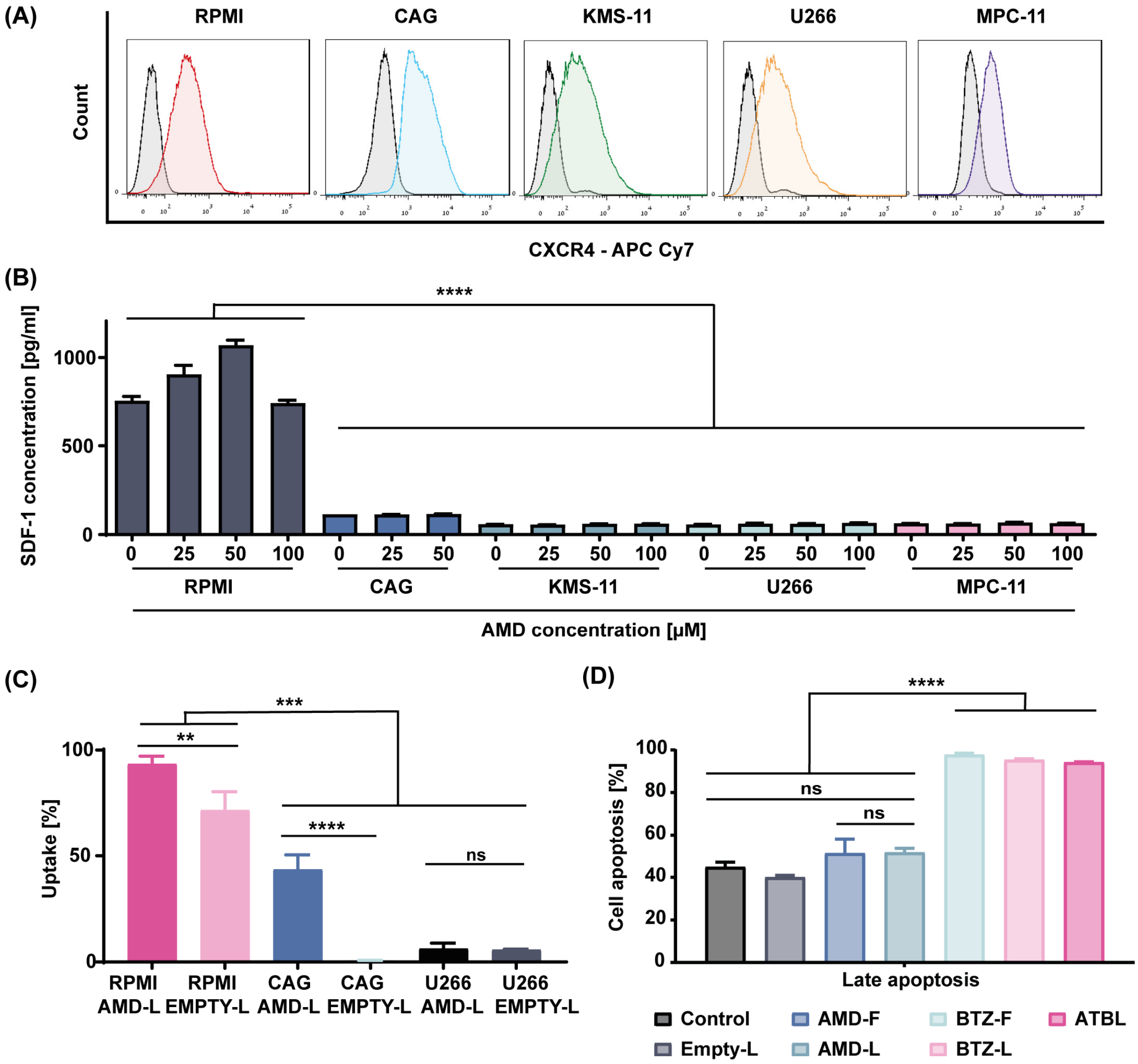

(A)
RPMI
CAG
KMS-11
U266
MPC-11
Count
CXCR4 - APC Cy7
(B)
****
SDF-1 concentration [pg/ml]
1000
500
0
0 25 50 100 0 25 50 0 25 50 100 0 25 50 100 0 25 50 100
RPMI
CAG
KMS-11
U266
MPC-11
AMD concentration [µM]
(C)
***
**
****
ns
Uptake [%]
100
50
0
RPMI AMD-L
RPMI EMPTY-L
CAG AMD-L
CAG EMPTY-L
U266 AMD-L
U266 EMPTY-L
(D)
****
ns
ns
Cell apoptosis [%]
100
80
60
40
20
0
Late apoptosis
Control
Empty-L
AMD-F
AMD-L
BTZ-F
BTZ-L
ATBL

Figure S2: ATBL uptake is CXCR4 dependent: (A) Representative flow cytometry histograms of CXCR4 expression across various MM cells are shown. (B) The evaluation of SDF-1 secretion from several MM cells was carried out by ELISA (n=3/group). (C) In vitro cellular uptake at 90 min time-point of Cy5 labeled AMD-L ($4.86 \pm 1.04 \times 10^{11}$ liposomes/ml) compared to EMPTY-L ($4.56 \pm 1.19 \times 10^{11}$ liposomes/ml) in MM RPMI, CAG and U266 cell lines was carried out by flow cytometry (n=3/group). (D) The late apoptosis profile of CAG cell line was assessed by flow cytometry 48 hrs after cells were cultured with different treatments as indicated in the figure (n=3/group). Results are presented as mean ± SD. One-way ANOVA was used for the statistical analysis of B, C and D with multiple comparisons test. adjusted p-value; **$p < 0.01$, ***$p < 0.001$, ****$p < 0.0001$; ns, not significant; MM, multiple myeloma; RPMI, RPMI8226; ATBL, AMD targeted bortezomib liposomes; BTZ-F, bortezomib free drug; BTZ-L, bortezomib liposomes; AMD-F, AMD3100 free drug; AMD-L, AMD3100 liposomes; EMPTY-L, empty non-targeted liposome; Control, vehicle.

Figure S3

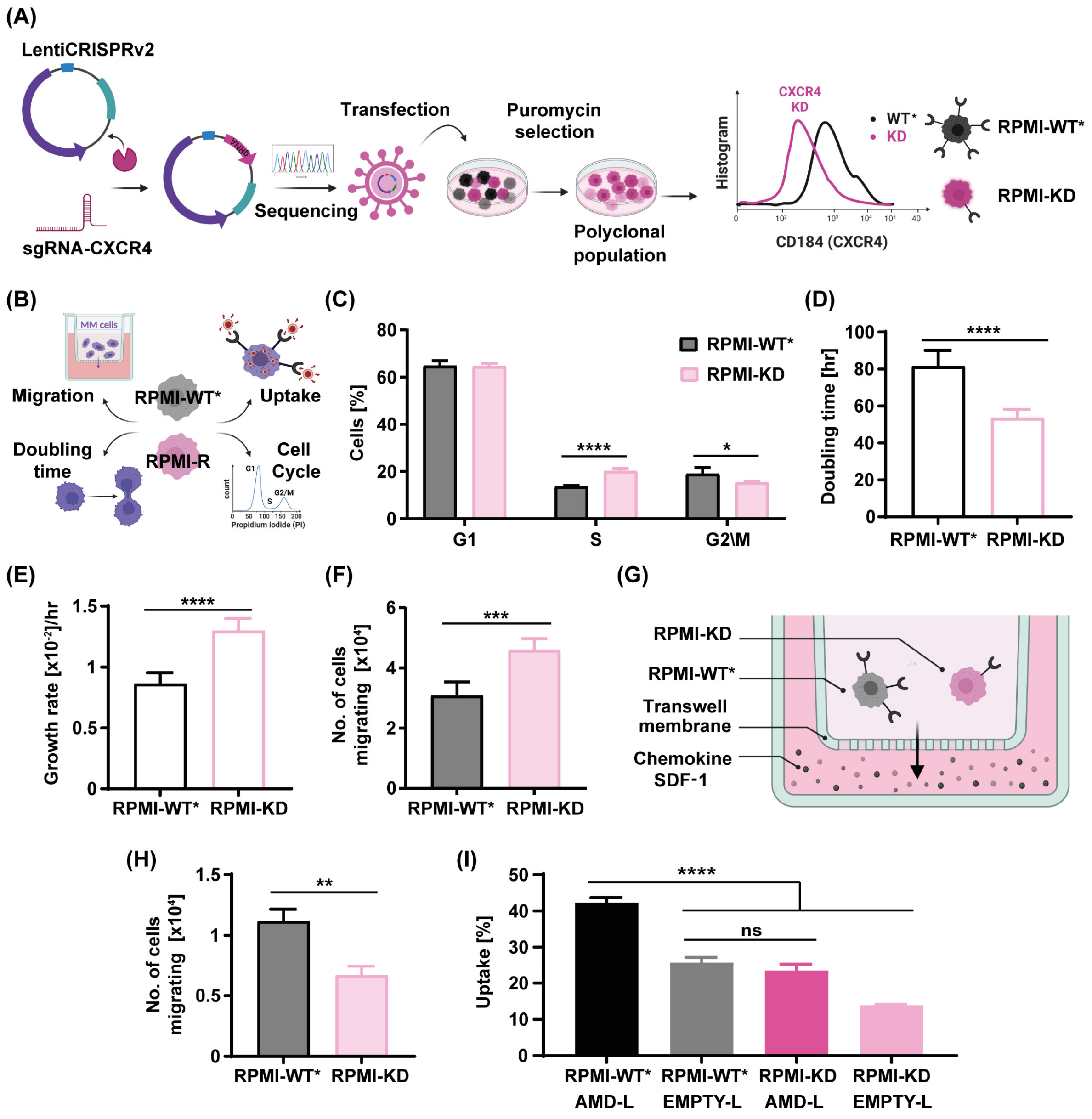

(A)
LentiCRISPRv2
sgRNA-CXCR4
Sequencing
Transfection
Puromycin selection
Polyclonal population
CXCR4 KD
WT*
KD
Histogram
CD184 (CXCR4)
RPMI-WT*
RPMI-KD
(B)
MM cells
Migration
RPMI-WT*
Uptake
Doubling time
RPMI-R
Cell Cycle
Propidium iodide (PI)
(C)
Cells [%]
RPMI-WT*
RPMI-KD
****
*
G1
S
G2\M
(D)
Doubling time [hr]
****
RPMI-WT*
RPMI-KD
(E)
Growth rate [x10-2]/hr
****
RPMI-WT*
RPMI-KD
(F)
No. of cells migrating [x10⁴]
***
RPMI-WT*
RPMI-KD
(G)
RPMI-KD
RPMI-WT*
Transwell membrane
Chemokine SDF-1
(H)
No. of cells migrating [x10⁴]
**
RPMI-WT*
RPMI-KD
(I)
Uptake [%]
****
ns
RPMI-WT* AMD-L
RPMI-WT* EMPTY-L
RPMI-KD AMD-L
RPMI-KD EMPTY-L

Figure S3: Generation and characterization of RPMI CXCR4 knockdown cells. (A) A schematic illustration of RPMI cells knocked down for CXCR4 (RPMI-KD) generated using the CRISPR-Cas9 methodology. (B) A schematic illustration of the experimental design of MM RPMI-KD and their wild-type counterpart (RPMI-WT*) cells tested for cell proliferation and migration. (C) Cell cycle was analyzed using flow cytometry (n=4/cell line). (D-F) Cell doubling time (n=6/group) (D); cell growth rate (n=6/group) (E); and cell migration (n=4-5/group) (F) were plotted using graph bars (G) A schematic illustration of the transwell migration assay using MM RPMI-KD and RPMI-WT* cells migrating in response to the SDF-1 chemokine. (H) The number of migrated RPMI-KD and RPMI-WT* cells was assessed by flow cytometry (n=3/group). (I) An in vitro cellular uptake of MM RPMI-KD and RPMI-WT* cells, at 90 min time-point was assessed using rhodamine labeled AMD-L ($4.86 \pm 1.04 \times 10^{11}$ liposomes/ml) compared to EMPTY-L ($4.56 \pm 1.19 \times 10^{11}$ liposomes/ml) (n=3/cell line). Results are presented as mean ± SD. Two-way ANOVA was used for the statistical analysis of C and G, with multiple comparisons test. Two-tailed unpaired Student's t-test was used for the statistical analysis of D-F and H, adjusted p-value; *$p < 0.05$, **$p < 0.01$, ***$p < 0.001$, ****$p < 0.0001$. MM, multiple myeloma; RPMI-KD, RPMI8226 CXCR4 knockdown cells; RPMI-WT*, RPMI8226 wild type cells that underwent the same genetic manipulation as the RPMI-KD cells, but without the inclusion of the guide RNA; AMD-L, AMD3100 liposomes; EMPTY-L, empty non-targeted liposome.

Figure S4

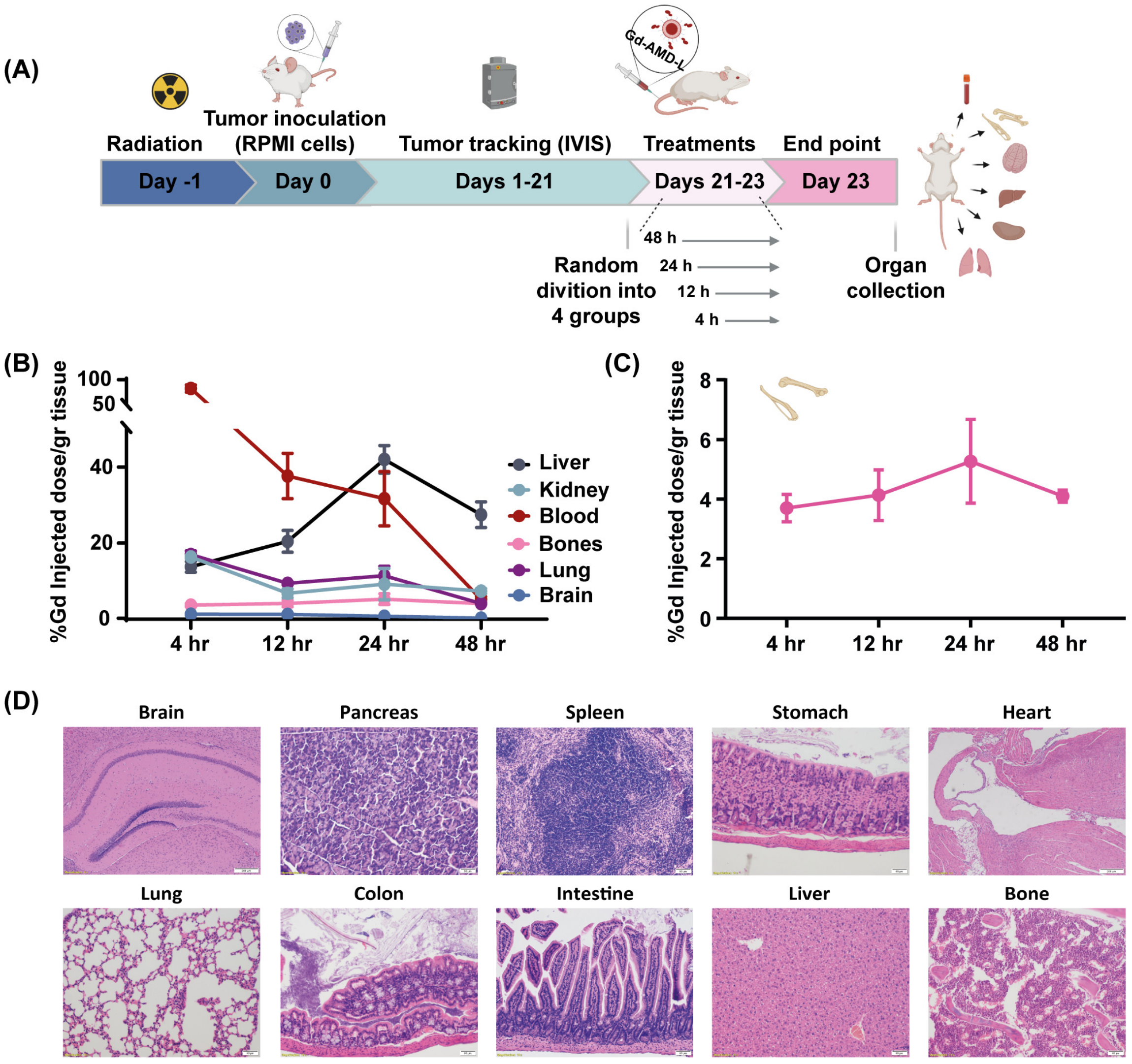
(A)
Radiation
Tumor inoculation (RPMI cells)
Tumor tracking (IVIS)
Treatments
End point
Gd-AMD-L
Day -1
Day 0
Days 1-21
Days 21-23
Day 23
48 h
24 h
12 h
4 h
Random divition into 4 groups
Organ collection
(B)
%Gd Injected dose/gr tissue
100
50
40
20
0
4 hr
12 hr
24 hr
48 hr
Liver
Kidney
Blood
Bones
Lung
Brain
(C)
%Gd Injected dose/gr tissue
8
6
4
2
0
4 hr
12 hr
24 hr
48 hr
(D)
Brain
Pancreas
Spleen
Stomach
Heart
Lung
Colon
Intestine
Liver
Bone

Figure S4: ATBL effectively reaches the bones with minor toxicities. (A) A schematic illustration of the experimental design: Eight-week-old SCID mice were systemically irradiated (250 rad) and after 24 hrs MM RPMI cells ($5x10^6$) were intravenously injected. After 21 days, when sufficient tumor burden was detected by IVIS, AMD-L containing gadolinium (Gd) ($8.10 \pm 1.74x10^{13}$ liposomes/kg) were intravenously administrated. (B-C) Organs were collected at the indicated time points (n=3 mice/group) and analyzed as the injected %Gd normalized to the organ weight, using ICP (B), where the Gd concentration in the bones was also plotted separately (C). (D) In a separate experiment, 8-week-old BALB/c mice were treated with ATBL ($1.95 \pm 0.43x10^{13}$ liposomes/kg), BTZ-F (1 mg/kg) or control once a week for 4 weeks (n=5 mice/group). At endpoint, histological organ sections of brain, pancreas, spleen, stomach, heart, lung, colon, intestine, liver and bone were stained with H&E to identify changes in tissue structure. Scale bar, 50 µm. Results are presented as mean ± SD. MM, multiple myeloma; RPMI, RPMI8226; ATBL, AMD targeted bortezomib liposomes; BTZ-F, bortezomib free drug; AMD-L, AMD3100 liposomes; Control, vehicle.

Figure S5

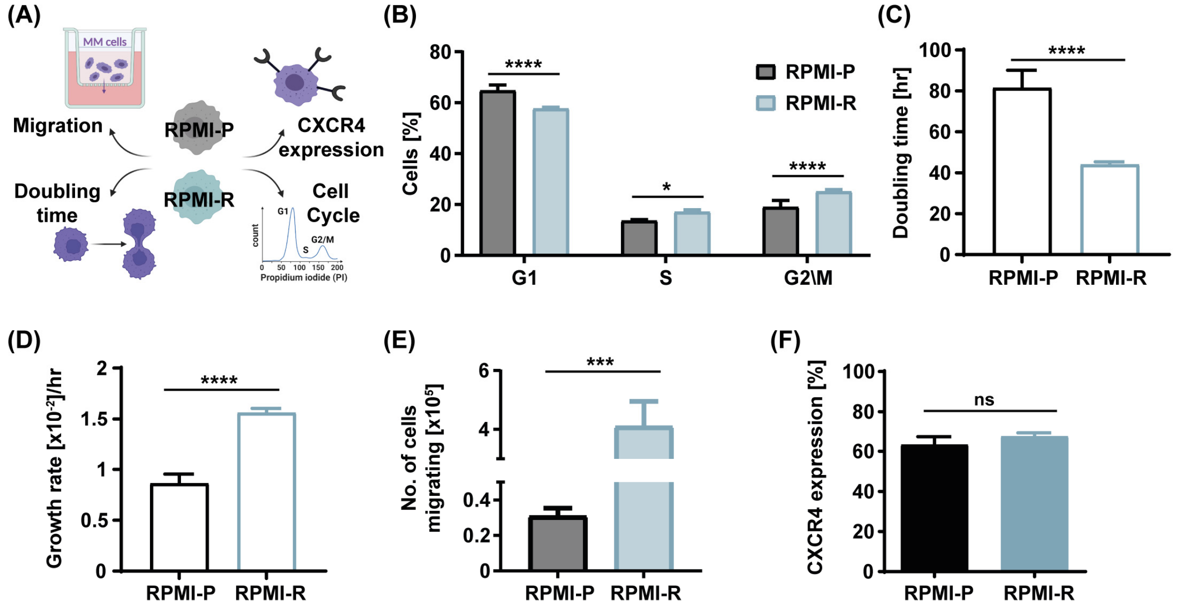

Figure S5: Characterization of RPMI cells resistant to BTZ therapy. (A) A schematic illustration of the experimental design testing the proliferation and migration of RPMI MM cells resistant and sensitive to bortezomib (RPMI-R and RPMI-P, respectively). (B) Cell cycle analysis was assessed using flow cytometry (n=4/group); (C-E) Cell doubling time (n=6/group) (C); growth rate (n=6/group) (D); and cell migration (n=4/group) (E) are shown by bar graphs. (F) CXCR4 expression in MM RPMI-R and RPMI-P cells was assessed using flow cytometry (n=3/group). Results are presented as mean ± SD. Two-way ANOVA was used for the statistical analysis of B, with multiple comparisons test, and Two-tailed unpaired Student's t-test was used for the statistical analysis of C-F, adjusted p-value; *p < 0.05, ***p < 0.001, ****p < 0.0001. ns, not significant; MM, multiple myeloma; BTZ, bortezomib; RPMI-R, RPMI8226 cells resistant to BTZ; RPMI-P, parental RPMI8226.

Figure S6

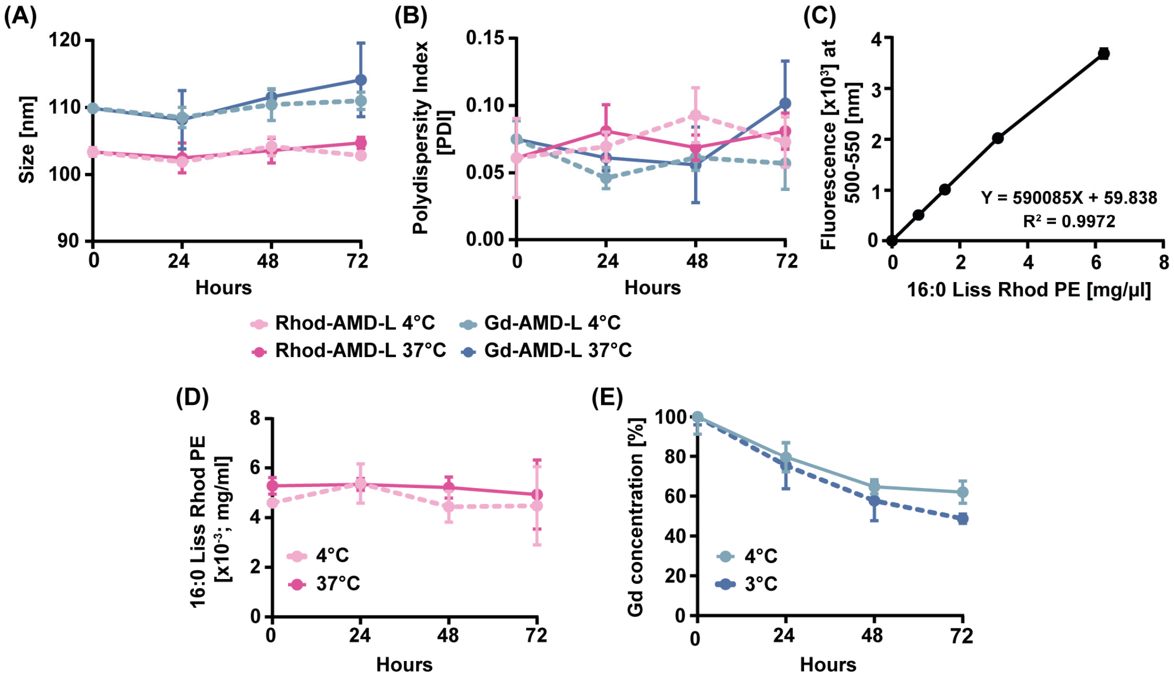

Figure S6: Stability characterization of Rhod-AMD-L and Gd-AMD-L. Liposomes were dialyzed at 4 °C and 37 °C at 4 time points over 72 hrs. (A-B) Liposome mean diameter (nm) (n=3/group) (A), and Polydispersity index (PDI) (n=3/group) (B) measured by Zetasizer. (C) A calibration curve of known 16:0 Liss Rhod PE lipid concentrations at 500-550 nm wavelength (n=3/16:0 Liss-Rhod PE concentration). (D) Quantification of the 16:0 Liss Rhod PE lipid concentration in Rhod-AMD-L (n=3/group). (E) Gd release profile of gadolinium from Gd-AMD-L over 72 hrs (n=3/group). Results are presented as mean ± SD. Rhod, rhodamine; Gd, gadolinium; Rhod-AMD-L, rhodamine labeled AMD targeted liposomes; Gd-AMD-L, AMD targeted gadolinium liposomes.
